# Silent catalytic promiscuity in the high-fidelity terpene cyclase δ-cadinene synthase†
†Electronic supplementary information (ESI) available: General experimental procedures, enzyme preparation and purification, kinetics data, gas chromatograms, mass spectra and NMR spectra. See DOI: 10.1039/c8ob02821d


**DOI:** 10.1039/c8ob02821d

**Published:** 2019-01-17

**Authors:** Marianna Loizzi, David J. Miller, Rudolf K. Allemann

**Affiliations:** a School of Chemistry , Cardiff University Main Building , Park Place , Cardiff , CF10 3AT , UK . Email: allemannrk@cardiff.ac.uk

## Abstract

δ-Cadinene synthase (DCS) is a high-fidelity sesquiterpene synthase that generates δ-cadinene as the sole detectable organic product from its natural substrate (*E*,*E*)-FDP. Previous work with this enzyme using substrate analogues revealed the ability of DCS to catalyse both 1,10- and 1,6-cyclisations of substrate analogues. To test whether this apparent promiscuity was an artefact of alternate substrate use or an inherent property of the enzyme, aza analogues of the proposed α-bisabolyl cation intermediate were prepared since this cation would be formed after an initial 1,6-cyclisation of FDP. In the presence of 250 μM inorganic disphosphate both (*R*)- and (*S*)-aza-bisaboyl cations were potent competitive inhibitors of DCS (*K*_i_ = 2.5 ± 0.5 mM and 3.44 ± 1.43 μM, respectively). These compounds were also shown to be potent inhibitors of the 1,6-cyclase amorpha-4,11-diene synthase but not of the 1,10-cyclase aristolochene synthase from *Penicillium roquefortii*, demonstrating that the 1,6-cyclase activity of DCS is most likely an inherent property of the enzyme even when the natural substrate is used and not an artefact of the use of substrate analogues.

## Abstract 2

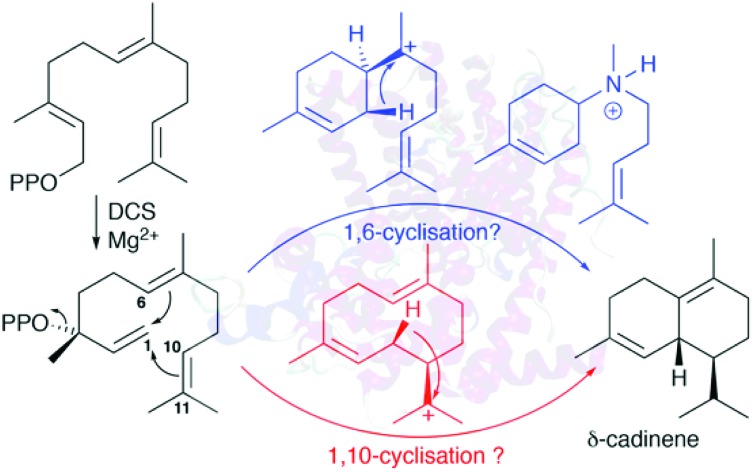
Aza-analogues of carbocations inhibit δ-cadinene synthase: 1,6-cyclisation.

## Introduction

Terpene synthases catalyse some of the most complex reactions in the natural world. From a small pool of isoprenyl diphosphates they generate a myriad of hydrocarbons and alcohols that are often processed into thousands of terpenoids with diverse biological activities with many potential applications for instance as agrochemicals or therapeutic agents.^1^

**Scheme 1:**
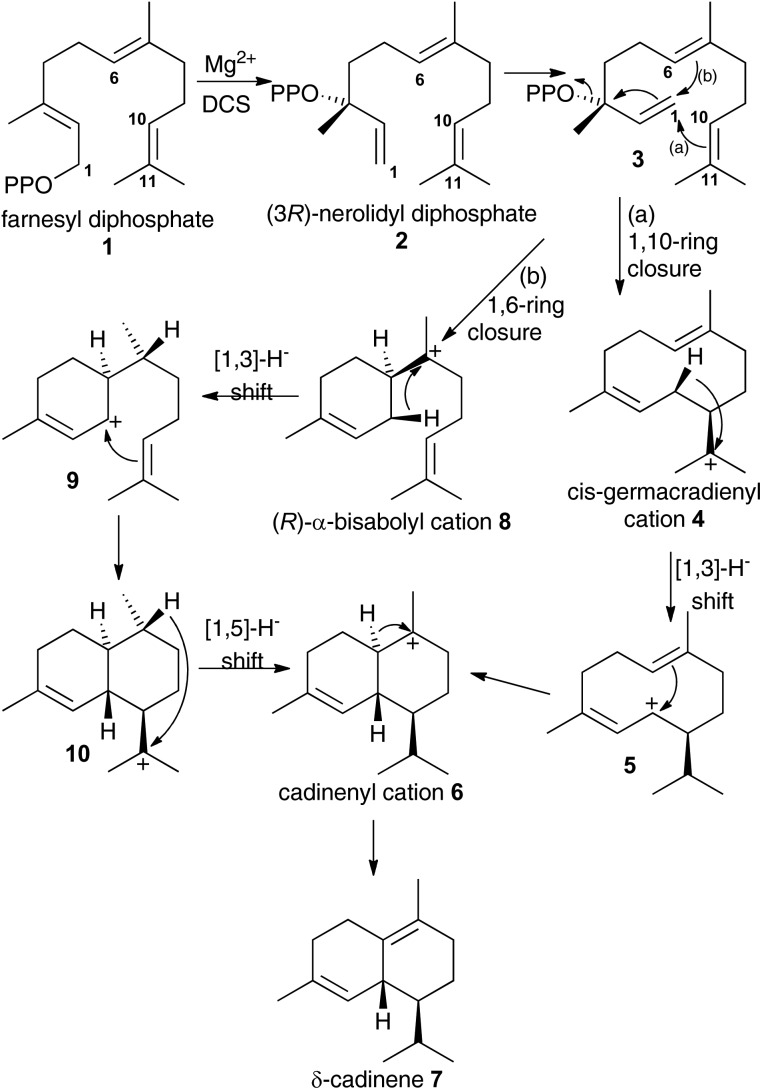
Possible chemical steps for DCS catalysed production of δ-cadinene from FDP (**1**).

The details of terpene synthase chemistry^2^ have been investigated by site directed mutagenesis and with non-natural amino acids,^3^ analogues of substrates,^4^ and putative reaction intermediates,^5^ X-ray crystallography,2b,c,^6^ and computational modelling.2d,^7^ Together these investigations revealed a fascinating, yet still incomplete picture. A series of X-ray crystal structure of aristolochene synthase from *Aspergillus terreus* in both closed and open conformations along with complexes containing the complete substrate (or analogue), diphosphate anion and/or Mg^2+^ co-factors^8^ revealed the physical steps of the catalytic cycle. Binding of a Mg^2+^-ion is followed by coordination of the prenyl diphosphate substrate and a second Mg^2+^ ion; coordination of a third Mg^2+^ ion triggers active site closure to form the Michaelis complex.^8^ Diphosphate cleavage is then triggered to form an initial carbocation and the hydrophobic active site shelters this high energy intermediate from bulk solvent.2b,c The active site, lined with hydrophobic and aromatic amino acid residues then steers the initial carbocation through a series of ring closures and rearrangements prior to quench of the final carbocation either by proton loss or nucleophilic attack by water.2b,c,6e Usually this is tightly controlled by the enzyme, with a single enantiomer dominating the product pool whereby several rings and stereocentres are often generated in a single chemical step from an achiral precursor. Control of this process is thought to arise from a product-like active site contour in combination with direction of carbocation location in the intermediates through the negative charge on the diphosphate anion and aromatic amino acid side chains that can stabilise carbocations at certain locations through cation–π interaction.2b,c,4a A small subset of terpene synthases, on the other hand, exhibit significant promiscuity, presumably through having a less structured and/or flexible active site that allows the intermediates to sample a large number of reactive conformations prior to final carbocation quench. For example, δ-selinine synthase and γ-humulene synthases from *Abies grandis* generate 34 and 52 products from farnesyl diphosphate (**1**), respectively.^9^ Terpene synthases have been postulated to evolve through such promiscuous intermediates prior to further evolution into high-fidelity synthases.3*e* The modern δ-cadinene synthase (DCS) from *Gossypium arboreum* is a high-fidelity sesquiterpene synthase that catalyses the formation of the bicyclic hydrocarbon (+)-δ-cadinene (**7**),^10^ the first committed step in the biosynthesis of the phytoalexin gossypol.^11^ The catalytic domain is situated in the C-terminal domain and adopts the α-helical fold domain, typical of class 1 terpene synthases.2b,c,^12^ It contains the conserved aspartate rich motif D^307^DTYD^311^ on helix D, but instead of the usual characteristic NSE/DTE Mg^2+^ binding motif, DCS has a second aspartate rich motif D^451^DVAE^455^ on helix H.6*e* Despite only generating a single detectable hydrocarbon product, extensive mechanistic analysis of the DCS-catalysed reaction pathway has not unambiguously defined the chemical steps of its catalytic cycle. Moreover, conversion of fluorinated and stereochemically altered FDP analogues with DCS revealed an underlying mechanistic promiscuity with products arising from an initial 1,10-, 1,6- or 1,11-ring closure depending upon the substrate analogue used (*vide infra*).^12^ Two chemical mechanisms remain plausible for the formation of δ-cadinene from FDP (Scheme 1). Both pathways involve initial formation of (3*R*)-nerolidyl diphosphate ((3*R*)-NDP, (**2**)) as an enzyme-bound intermediate. In pathway (a), a 1,10-macrocyclisation occurs to generate *cis*-germacradienyl cation (**4**). A subsequent [1,3]-hydride shift is followed by a 1,6-electrophilic ring closure to cadinenyl cation (**6**), from which δ-cadinene (**7**) is formed after proton loss from C6. In pathway (b), a 1,6 ring-closure of **2** is followed by a [1,3]-hydride shift from C1 to C7; subsequently a second ring closure and a [1,5]-hydride shift lead to cadinenyl cation, an intermediate common to both pathways. In previous work, using substrate analogues we were unable to definitively rule out pathway (b) and indeed when 6-fluorofarnesyl diphosphate (6F-FDP) was used as a substrate analogue it proved to be a potent inhibitor (*K*_i_ = 2.4 μM), giving no detectable pentane-extractable products when incubated with DCS. This result is consistent with an initial 1,6-cyclisation pathway since it would be expected to undergo 1,10-ring closure and give an abortive product rather than inhibit the enzyme in the latter scenario. On the other hand, 2-fluorofarnesyl diphosphates (2F-FDP) and 10-fluorofarnesyl diphosphate (10F-FDP) gave products arising from 1,10- and 1,11 ring-closures, respectively, consistent with an initial 1,10-ring closure mechanism.^12^

Hence examination of the catalytic mechanism of DCS using FDP analogues has led to inconclusive, yet intriguing results, showing that this enzyme has the potential to use alternative reaction pathways. Yet the question arises, is this simply an artefact of the substrate used or is this an inherent property of the enzyme? The work described here provides alternative mechanistic data for the DCS-catalysed transformation of FDP to δ-cadinene using aza-analogues of putative carbocation intermediates. Although the highly unstable carbocationic intermediates formed during terpene synthase catalysis, cannot be isolated, it is possible to replace the sp^2^ hybridised carbocationic carbon of a given intermediate with an sp^3^ hybridised nitrogen in an amine analogue or with a sp^2^ hybridised nitrogen in an iminium ion. Although the tetrahedral tertiary ammonium ions inherently are imperfect geometric analogues of the planar carbocations, these aza-terpenoids are thought to mimic the topological and electrostatic properties of carbocations generated by these enzymes.^5^ However, since they cannot be processed by the enzyme, they often act as tightly bound competitive inhibitors of terpene synthases.5c,^13^

Hence, the use of strategically designed aza-analogues may enable the disentanglement of the possible reaction mechanisms catalysed by DCS. Here we report the stereoselective synthesis of the two enantiomers of aza-bisabolyl cation and their kinetic evaluation as inhibitors of DC*S*. Comparison of their effect upon catalysis by AS and amporpha-4,11-diene synthase (ADS), enzymes that follow 1,10- and 1,6-ring-closure mechanisms, validate the result that DCS has inherent 1,6- as well as 1,10 ring closure activity.

## Results and discussion

**Fig. 1:**
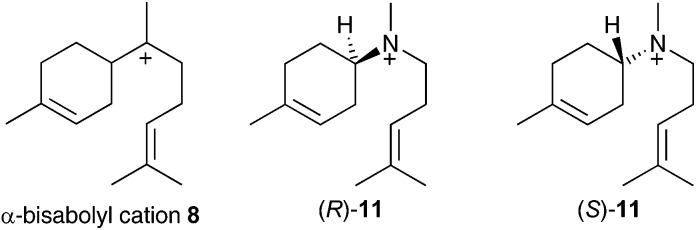
Chemical structures of the α-bisabolyl cation (**8**) and corresponding aza analogues (*R*)-**11** and (*S*)-**11**.

If α-bisabolyl cation **8** is a reaction intermediate on the pathway to δ-cadinene (**7**), one or both of enantiomeric aza-analogues of **11** (Fig. 1) should act as competitive inhibitors of DCS.

**Scheme 2:**
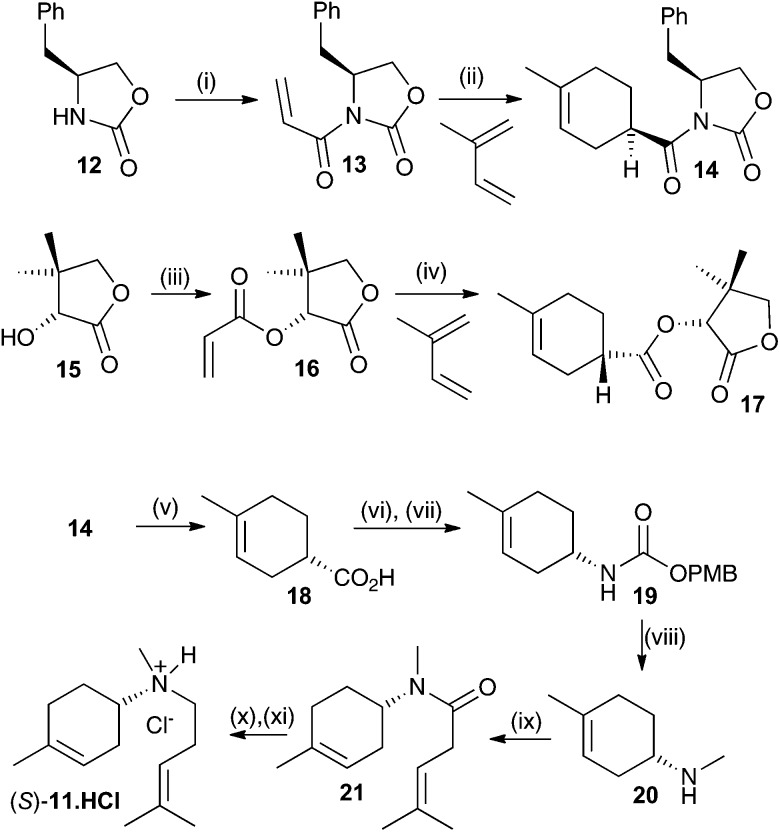
Synthesis of aza-analogues (*R*)- and (*S*)-**11**. Reagents and conditions: (i) acryloyl chloride, BuLi, THF, 35%. (ii) Et_2_AlCl, CH_2_Cl_2_, –100 °C, 54%. (iii) Acryloyl chloride, NEt_3_, CH_2_Cl_2_, 60%. (iv) TiCl_4_, CH_2_Cl_2_, –10 °C, 84%. (v) LiOH, THF, H_2_O, MeOH, 50 °C, 99%. (vi) DPPA, NEt_3_. (vii) *p*-Methoxybenzyl alcohol, toluene, 60% over two steps. (viii) LiAlH_4_, Et_2_O, 50%, (ix) 4-methylpent-3-enoic acid, EtNPr^i^_2_, HBTU, DMF, 70%. (x) LiAlH_4_, Et_2_O. (xi) HCl in Et_2_O, 55% two steps.

Both enantiomers of **11** have previously been prepared.14*a* Here we report an alternative synthesis that is more concise and avoids the use of harsh reaction conditions. Key to the synthesis of both enantiomers is an enantioselective synthesis of the two enantiomers of carboxylic acid **18** (Scheme 2). This was achieved through asymmetric Diels–Alder reaction of an acrylate derivatised with a chiral auxiliary with a butadiene.^15^ Oxazolidin2-one **12** was alkylated with acryloyl chloride after deprotonation with *n*-butyl lithium with 35% yield. The resulting ester **13** was then subjected to an asymmetric Diels–Alder reaction with 2-methylbutadiene.^15^ The enantioselectivity and yield were optimal at –100 °C in CH_2_Cl_2_ (52%, ee >95%, de >95% (see ESI† for details). This produced the key compound to generate the *S* enantiomer of the aza-analogue **11**. The equivalent *R* configured ester was generated using d-pantolactone (**15**) as a chiral auxiliary.^15^ After alkylation with acryloyl chloride, using NEt_3_ as the base in CH_2_Cl_2_, diester **16** was isolated in 60% yield. Again, an asymmetric Diels–Alder reaction with 2-methylbutadiene was carried out, this time at –10 °C in CH_2_Cl_2_ using TiCl_4_ as a Lewis acid catalyst yielding the *R* ester in 84% yield (ee = 92% and de = 97%).^16^ The latter procedure was in fact optimal for both enantiomers but due to the high cost of l-pantolactone not used for bulk preparation for the *R*-enantiomer of **18**. Optical purity of all subsequent compounds was checked using chiral GC, HPLC and/or polarimetry and in all cases no loss of optical purity was detected in later synthetic steps.

Both syntheses now proceeded in identical manner and Scheme 2 only illustrates the synthesis of the *S*-enantiomer of **11**. Hydrolysis of **14** using LiOH in an equivolume mixture of THF, water and methanol for 1 h at 50 °C gave carboxylic acid **18** in near quantitative yield. **18** was converted to *p*-methoxybenzyl urethane derivative **19** by treatment with diphenylphosphorylazide (DPPA) followed by a Curtius rearrangement in the presence of *p*-methoxybenzyl alcohol, which proceeded with strict retention of stereochemistry.15*b* The overall yield of the urethane product **19** was 60% over the two steps. Final conversion to (*S*)-**11** was achieved first through reduction with LiAlH_4_ in anhydrous Et_2_O (50%) then HBTU mediated coupling to 4-methypent-3-enoic acid (70%) followed by a second reduction with LiAlH_4_ in Et_2_O. To prevent air oxidation upon storage the product was converted to its hydrochloride salt with HCl in ether, yielding (*S*)-**11**·**HCl** in 55% yield over the final two steps. The optical purity of (*S*)-**11** was estimated to be ≥98% by comparison with previously reported data.14*a* Similar results were obtained for the synthesis of (*R*)-**11**.

**Scheme 3:**
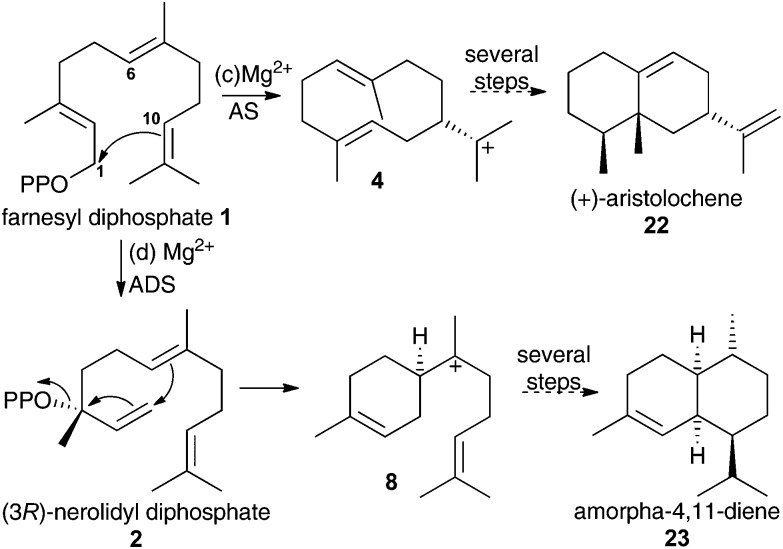
Initial catalytic chemical steps leading to (c) (+)-aristolochene (**22**) and (d) amorpha-4,11-diene (**23**) follow 1,10- and 1,6-cyclisation of FDP, respectively.

To validate any results obtained for these compounds as inhibitors of DCS, they were tested as inhibitors of aristolochene synthase from *Penicillium roqueforti* (AS) and amorpha-4,11-diene synthase (ADS). These two enzymes are known to proceed *via* 1,10- and 1,6-cyclisations of the initial carbocation during their catalytic cycle (Scheme 3).1*g* Hence aza-bisabolyl cations **11** should act as poor inhibitors of AS and potent inhibitors of ADS, as they closely resemble a reaction intermediate in the latter case only.

Recombinant AS and ADS were prepared and purified according to previously published procedures^17^,^18^ and both (*R*)-**11** and (*S*)-**11** were tested as inhibitors using a standard radiolabelled assay involving conversion of tritium labelled FDP by each enzyme and scintillation counting of the pentane extractable products.5*b* Terpene synthases are known to efficiently bind cation-PP_i_ pairs and inhibition was assessed both in the presence and absence of 250 μM diphosphate (Table 1). Synergistic inhibition of aza-analogues **11** with diphosphate has been observed previously for a variety of other terpene synthases.5d,13c,^14^

**Table 1:** Kinetic data for inhibition of ADS, AS and DCS by (*R*)-(**11**) and (*S*)-**11**. Uninhibited kinetic data for each enzyme: ADS *K*_M_ = 2 ± 0.15 μM *k*_cat_ = 1.19 × 10^–2^ ± 52 × 10^–5^ s^–1^. AS *K*_M_ = 2.42 ± 0.11 μM, *k*_cat_ = 1 × 10^–2^ ± 2 × 10^–5^ s^–1^. DCS-His_6_*K*_M_ = 0.58 ± 11 μM *k*_cat_ = 1.26 × 10^–3^ ± 5 × 10^–6^ s^–1^

Enzyme	Aza-analogue	*K*_i_ (μM) (+250 μM PP_i)_	*K*_i_ (μM)
ADS	(*S*)-**11**	1.5 ± 0.5	25 ± 5
	(*R*)-**11**	3.7 ± 1.9	50 ± 17
AS	(*S*)-**11**	255 ± 23	295 ± 23
	(*R*)-**11**	489 ± 62	472 ± 48
DCS	(*S*)-**11**	3.44 ± 1.43	273 ± 77
	(*R*)-**11**	2.5 ± 0.5	1700 ± 300

Kinetic data were fitted by non-linear regression to the Michaelis–Menten equation (*v*_0_ = *k*_cat_[E][S]/(*K*_M_ + [S]). The mode of inhibition was determined by inspection of double reciprocal plots and observed to be competitive in all cases where inhibition was significant at low concentrations of **11**. *K*_I_ was determined from a plot of inhibitor concentration *versus K*′_M_/(*k*_cat_[E]) where *K*′_M_ = *K*_M_(1 + [I]/*K*_I_).

The inhibition data for AS and ADS validate both of these compounds as valuable mechanistic probes for the present investigation since they are poor inhibitors of AS and potent inhibitors of ADS. PP_i_ had little effect on the ability to inhibit AS (*K*_I_ > 200 μM in both the presence and absence of PP_i_ for AS). Both enantiomers of **11** acted as competitive inhibitor of ADS, showing that they are able to compete effectively with the natural substrate FDP at the active site. As these aza-compounds cannot be turned over by ADS, these result support the intermediacy of an α-bisabolyl cation in the biosynthesis of amorpha-4,11-diene, in agreement with the findings of Picaud *et al.*18*b* who used deuterated farnesyl diphosphate and deuterium exchange experiments to suggest that the *R*-enantiomer of the α-bisabolyl cation is the sole intermediate formed in the biosynthesis of amorpha-4,11-diene. Therefore, only the *R* enantiomer of **11** would be expected inhibit ADS; however, if the *S*-enantiomer was a slightly more potent inhibitor (*K*_i_ = 50 μM for (*R*)-**11***versus* 25 μM for (*S*)-**11**) Table 1. This is consistent with a flexible model for sesquiterpene active sites, according to which an active site can accommodate a variety of intermediates of different shape and charge distribution without being rigidly complementary to a single intermediate or transition state species. For example, work by Cane *et al.* showed that both enantiomers of the aza-analogue **11** were equally effective inhibitors of trichodiene synthase.14*a* It is also notable that the presence of PP_i_ enhanced inhibition of ADS by both enantiomers, improving the *K*_i_ approximately 20-fold (*K*_i_ = 1.5 and 3.7 μM for the *S* and *R* enantiomers respectively) demonstrating that the active site of ADS prefers a cation–anion pair in its active site.5d,^13^

Recombinant DCS was generated with a C-terminal hexahistidine tag (DCS-His_6_) as previously described.^19^ Inhibition assays were carried out using the same protocol used for AS and ADS. Both aza analogues were found to be competitive inhibitors of DCS-His_6_ in the presence of PP_i_ but only poor inhibitors in its absence (Table 1). DCS clearly requires a cation–anion pair in its active site for effective inhibition by aza-analogues. Our results provide strong evidence for 1,6-cyclase activity for DCS.

## Conclusions

**Fig. 2:**
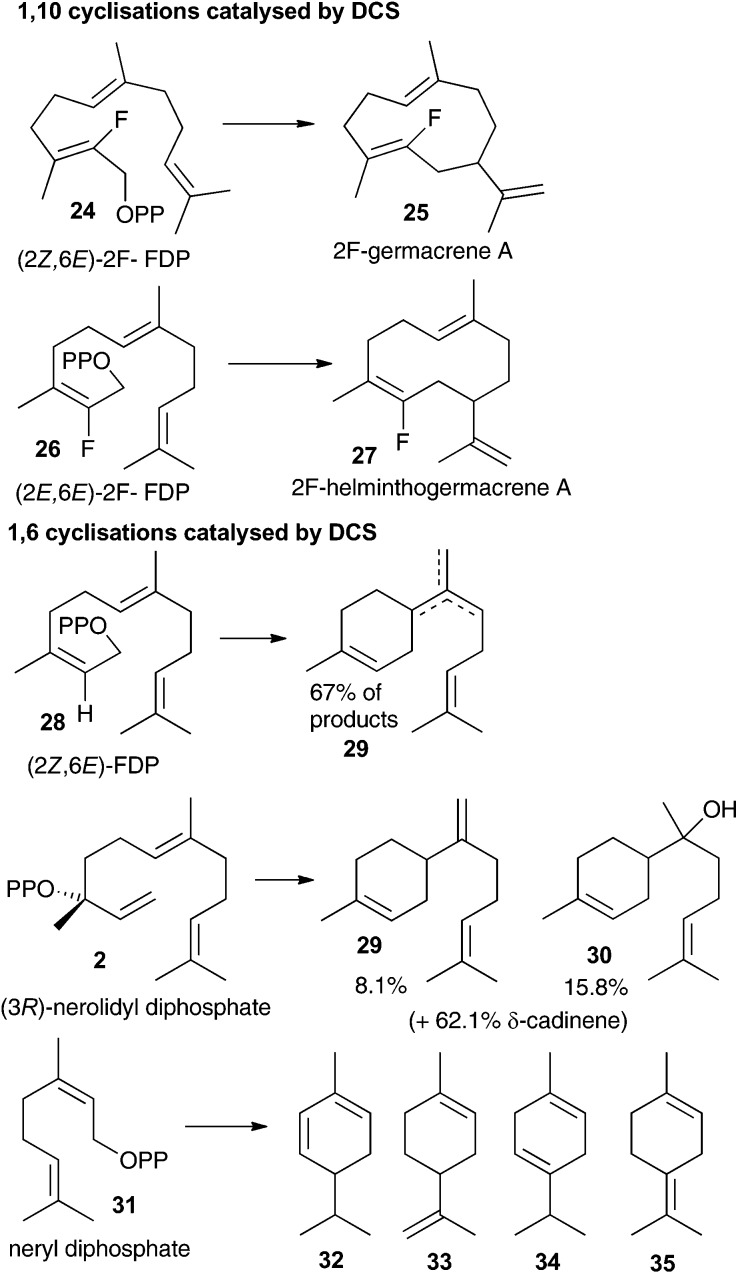
Summary of 1,10 and 1,6 cyclisation products generated through DCS catalysis.

The aza-bisabolyl cations **11** were potent competitive inhibitors of ADS, a 1,6-cyclase yet were much poorer inhibitors of PR-AS, a known 1,10-cyclase. When DCS was challenged with these aza-analogues in the presence of diphosphate anion they were potent inhibitors of the conversion of FDP to (+)-δ-cadinene (**7**), which would only be expected if DCS had a 1,6-cyclase activity. The use of a variety of substrate analogues possessing different stereochemistry and heteroatoms did not lead to clear results regarding whether DCS follow a 1,6 or 1,10 pathway.^12^ If the proposed initial isomerism of the substrate to nerolidyl diphosphate (**2**) was suppressed using a fluorine atom at C2 then a 1,10 cyclisation was observed (Fig. 2). 2-Fluorogemacrene A (**25**) was the DCS catalysed product from the transoid (2*Z*,6*E*)-2-fluorofarnesyl diphosphate (**24**) while the cisoid substrate analogue **26** gave the cisoid product 2F-helminthogermacrene A (**27**).^12^ However, in nearly every other case involving the use of substrate analogues with DCS, 1,6-cyclisation was observed at least in-part (Fig. 2).^12^ These results may simply reflect the use of different substrates rather than an inherent ability of DCS to catalyse the conversion of FDP to **7** along two distinct reaction paths.^20^ The observation that **11** acts as a competitive inhibitor of the DCS catalysed conversion of FDP provides strong evidence that DCS can efficiently use a 1,6-cyclisation pathway.

The fact that inorganic diphosphate led to a more tightly bound active site carbocation/diphosphate ion pair is consistent with previous work where often the active site recognises a cation-PPi pair more effectively than the cation alone.5b,c–7a,^14^ The fact that (*R*)-**11** acts as a weak inhibitor in the absence of PP_i_ is more difficult to explain. It was previously suggested that, in the DCS active site pocket, the alkenyl chain of (3*R*)-nerolidyl diphosphate (**2**) is ideally positioned to ensure the formation of the α-bisabolyl cation with an *R* configuration at C6 (Scheme 1).^12^ The C1–C7 hydride shift from **8** to **9** then occurs to the same *Si* face of C7 in cation **8**, therefore a (7*R*)-**9** formation is expected (Scheme 1). Hence the (*R*)-**11** should mimic better the α-bisabolyl cation generated by this enzyme, and therefore act as competitive inhibitor with higher binding affinity when compared with the *S*-enantiomer. The evidence that both enantiomers of **11** are equally as effective in the presence of PP_i_ is consistent with a permissive model of the active site structure, according to which an active site should accommodate a variety of rearranged intermediates of different shape and charge distribution without being rigidly complementary to a single intermediate. On the other hand, their lack of inhibitory effects on the 1,10-cyclase PR-AS shows that a major difference in the connectivity of the aza-analogue compared to the carbocation intermediate (*i.e.* bisabolyl cation rather than the 10-membered ring containing germacrenyl cation) renders them ineffective as inhibitors; hence the 1,6-cyclase activity of DCS postulated previously^12^ is intrinsic to the enzyme.

Terpene synthases can generate great structural and stereochemical complexity in one synthetic step and have therefore potential as powerful synthetic biocatalysts for the generation of many bioactive compounds.4f,18a,^21^,^22^ A clear understanding of the catalytic strategies employed by these enzymes can aid their redesign to produce nature-like compounds that are not found in the biosphere.^23^,^24^

## Experimental

General experimental procedures, enzyme preparation and purification are described in ESI† along with kinetics data, gas chromatograms, mass spectra and NMR spectra.

### (*R*)-4,4-Dimethyl-2-oxotetrahydrofuran-3-yl acrylate (**16**)

Freshly distilled propenoyl chloride (0.41 mL, 5 mmol) was added over 1 h to a stirred solution of (*R*)-pantolactone (500 mg, 3.84 mmol) and Et_3_N (583 mg, 5.76 mmol) in anhydrous CH_2_Cl_2_ (10 mL) at –24 °C. The resulting mixture was stirred for 5 h at –24 °C, and subsequently washed with aqueous 1 M HCl (10 mL). The aqueous phase was then extracted with CH_2_Cl_2_ (3 × 20 mL). The combined organic phases were washed with saturated NaHCO_3_ solution (3 × 20 mL), water (3 × 20 mL) and brine (3 × 20 mL). The organic phase was dried over MgSO_4_, concentrated under reduced pressure and the residue was purified by flash chromatography on silica (EtOAc : hexane 4 : 6) to yield the pure compound as a yellow oil (375 mg, 53%). *δ*_H_ (300 MHz, CDCl_3_) 6.48 (1 H, d, *J* = 17.5, CH*H*=C), 6.18 (1H, dd, *J* = 17.5, 10.5 Hz, C*H*=CH_2_), 5.93 (1H, d, *J* = 10.5 Hz, C*H*H=C), 5.40 (1H, s, C=OC*H*–O), 4.03 (2 H, s, C*H*_2_–OC=O), 1.17 (3 H, s, CH_3_), 1.08 (s, 3 H, CH_3_); *δ*_C_ (63 MHz, CDCl_3_) 174.7 (O*C*=OCHO), 172.5 (O*C*=OC=H_2_), 134.0 (H_2_*C*=CHC=O), 118.7 (H_2_C= *C*HC=O), 76.1 (OC=O*C*HO), 74.6 (O*C*H_2_CH), 40.2 (*C*–(CH_3_)_2_), 23.4 (CH_3_), 23.0 (CH_3_). *α*_D_ +10° (CH_2_Cl_2_, *c* = 17). Data are in agreement with previous work.15*a*

### (*R*)-4.4-Dimethyl-2-oxotetrahydrofuran-3-yl-(*S*)-4-methylcyclohex-3-ene-1-carboxylate (**17**)

To a solution of **16** (302 mg, 1.67 mmol) in anhydrous CH_2_Cl_2_ (5 mL) at –10 °C, TiCl_4_ (0.82 mL, 0.82 mmol, 1.0 M solution in CH_2_Cl_2_) was added, and the resulting solution was stirred under argon at –10 °C for 1 h. 2-Methylbutadiene (0.23 mL, 2.3 mmol) was then added over 5 min and the mixture was left stirring for 3 h at –10 °C. The reaction was quenched by addition of 10% Na_2_CO_3_ in water (5 mL). The aqueous phase was then extracted with CH_2_Cl_2_ (3 × 20 mL). The organic layers were combined, washed with H_2_O (3 × 10 mL), brine (3 × 10 mL), dried over anhydrous MgSO_4_, filtered and concentrated under reduced pressure. The residue was purified by flash chromatography on silica (EtOAc : hexane 2 : 8) to yield pure **17** as a colourless oil (353 mg, 85% yield). *δ*_H_ (300 MHz, CDCl_3_) 5.32 (1 H, s, OC*H*C=O,) and (1 H, br C*H*=), 3.98 (2 H, s, CHC*H*_2_O), 2.69–2.51 (1 H, m, C*H*–C=O), 2.19 (2 H, m broad, CHO–C*H*_2_C=), 1.98 (3 H, m, C*H*H–C*H*_2_), 1.73 (1H, m, –CH*H*–CH_2_), 1.58 (s, 3H, C*H*_3_C=), 1.13 (3H, s, C*H*_3_), 1.04 (3 H, s, C*H*_3_). *δ*_C_ (75 MHz, CDCl_3_) 174.7 (O*C*=OCHO), 172.5 (O*C*=OC=H_2_), 134.0 (HC= *C*CH_3_), 118.7 (H*C*=CCH_3_), 76.1 (OC=O*C*HO), 74.6 (O*C*H_2_C), 40.2 (*C*–(CH_3_)_2_), 38.9 (CH_2_C*H*–C=O), 28.9 (HC=C*C*H_2_), 27.7 (C=CH*C*H_2_), 25.2 (HC=CCH_2_*C*H_2_), 23.4 (C*C*H_3_), 23.0 (C*C*H_3_), 19.8 (*C*H_3_C=CH). LRMS (EI^+^) *m*/*z*: 252.13 (50%), 122.07 (20), 94.08 (100), 79.05 (30), 67.05 (10). *α*_D_ –51.3 (CHCl_3_, *c* = 1). Data are in agreement with previous work.^16^

### (*S*)-3-Acryloyl-4-benzyloxazolidin-2-one (**13**)

To a solution of (*S*)-4-benzyloxazolidin-2-one (1.00 g, 5.60 mmol) in anhydrous THF (12 mL) at –78 °C, *n*-BuLi (2.1 M in THF, 3.14 mL, 6.59 mmol) was added dropwise over 30 minutes and the mixture stirred for a further 3 h at –78 °C. Freshly distilled acryloyl chloride (557 mg, 6.16 mmol) was added dropwise over 20 minutes and the reaction stirred for 2 h at –78 °C. The reaction was then allowed to warm to room temperature overnight. The reaction was quenched with sat. NH_4_Cl (20 mL) and extracted with diethyl ether (3 × 30 mL). The organic layer was washed with water (3 × 40 mL), saturated aqueous NaHCO_3_ (3 × 40 mL), dried (MgSO_4_), filtered, and concentrated under reduced pressure. Flash chromatography on silica gel (hexane : ethyl acetate 6 : 4) afforded **13** as a colorless solid (452 mg, 35%). *δ*_H_ (CDCl_3_, 300 MHz) 7.45 (dd, 1H, *J* = 6.0, 18.0, C*H*=CH_2_), 7.23 (m, 5H, aromatic Hs), 6.54 (dd, 1H, *J*_H, H_ = 18.0, 18.0, CH*H*=CH_2_), 5.87 (dd, 1H, *J* = 9.0, 9.0, C*H*H=C), 4.68 (m, 1H, CHN), 4.14 (m, 2H, CH_2_O), 3.29 (dd, 1H, *J* = 9.0, 9.0, C=CH*H*Ph), 2.74 (dd, 1H, *J* = 12.0, 12.0, C*H*HPh) *α*_D_ –86° (CH_2_Cl_2_, *c* = 0.65). Data are in agreement with previous work.14*a*

### (*R*)-4-Benzyl-3-((*S*)-4-methylcyclohex-3-enecarbonyl)oxazolidin-2-one (**14**)

To a stirred solution of **13** (200 mg, 0.86 mmol) at –100 °C, were added 2-methylbutadiene (1.72 mL, 17.2 mmol) in anhydrous CH_2_Cl_2_ (5.0 mL) and Et_2_AlCl (1.2 mL, 1.5 eq.). The reaction was stirred at –100 °C for 30 min then the mixture was poured into ice cold aqueous hydrochloric acid (1 M, 20 mL). The mixture was extracted with CH_2_Cl_2_ (2 × 10 mL). The combined organic layers were dried over anhydrous MgSO_4_, filtered, and concentrated under reduced pressure. The product was purified by flash chromatography on silica (EtOAc : hexane : Et_3_N 92 : 7 : 1) to yield the Diels Alder adduct (**14**) as a white crystalline solid (139 mg, 54%). *δ*_H_ (300 MHz, CDCl_3_) 7.34–7.10 (5 H, m, ArCH), 5.36 (1 H, br., C*H*=C), 4.63 (1 H, dt, *J* = 16.6, 6.9, C*H*N), 4.23–3.95 (2 H, m, C*H*_2_O), 3.60 (1 H, t, *J* = 8.8, C*H*C=O), 3.20 (1 H, dd, *J* = 13.2, 3.3, CH*H*Ph), 2.70 (1 H, dd, *J* = 13.3, 9.5, C*H*HPh), 2.20–1.6 (6 H, m, C*H*_2_C*H*_2_CHC*H*_2_C=C), 1.64 (3H, s, *C*H_3_) *α*_D_ +79 (CH_2_Cl_2_, *c* = 1.4). LR-MS (EI^+^) *m*/*z*: 299.15 (100% M^+^), 300.16 (15), 269.06 (18), 267.07 (50), 232.10 (20), 178.08 (100), 146.07 (30), 140.03 (55), 122.07 (20), 91.00 (65), 63.00 (30). Data are in agreement with previous work.14*a*

### (*R*)- and (*S*)-4-Methylcyclohex-3-ene-1-carboxylic acid (**18**)

To a solution of **14** or **17** (0.32 mmol) in THF : MeOH : H_2_O (1 : 1 : 1, 1.5 mL), LiOH (67 mg, 1.6 mmol) was added, and the resulting mixture was vigorously stirred for 1 h at 50 °C. The reaction was then cooled to room temperature and concentrated under reduced pressure. The resulting slurry was dissolved in H_2_O (10 mL) and extracted with CH_2_Cl_2_ (3 × 5 mL). The resulting aqueous phase was acidified to pH = 2 at 0 °C with 15% HCl, extracted with a mixture of *n*-pentane : CH_2_Cl_2_ (98 : 2 3 × 10 mL), dried over anhydrous Na_2_SO_4_, and concentrated under reduced pressure to give **18** as a white powder (35 mg, 80%). *δ*_H_ (CDCl_3_, 300 MHz) *δ* 5.32 (1H, s, C*H*=), 2.54–2.39 (1 H, m, C*H*–COOH), 2.17 (2H, m, –C*H*_2_CH=C), 1.93 (3 H, m, C*H*H–C*H*_2_), 1.69 (1H, m, CH*H*–CH_2_), 1.59 (3H, s, C*H*_3_). *δ*_C_ (100 MHz, CDCl_3_) *δ* 182.3 (HO*C*=O), 133.8 (HC= *C*CH_3_), 119.0 (CH_2_–H*C*=CCH_3_), 39.0 (H*C*–COOH), 29.13 (*C*H_2_C=CH_3_), 27.3 (*C*H_2_CH=CCH_3_), 25.5 (*C*H_2_CH_2_C=CH), 23.5 (*C*H_3_). (*S*)-**18**: *α*_D_ –80.6 (CHCl_3_, *c* = 0.5); –106.4 (95% EtOH, *c* = 4). (*R*)-**18**: *α*_D_ +93 (CHCl_3_, *c* = 0.5); +105.5 (95% EtOH, *c* = 4). M.p. 82–92 °C. LRMS (EI^+^) *m*/*z* 140.06 (100%, M^+^), 136.06 (15), 125.05 (40), 122.06 (100), 95.87 (100), 94.06 (100), 93.07 (80), 79.04 (100), 77.03 (100), 68.06 (90), 67.04 (100) Data are in agreement with previous work.^15^,^16^

### (*R*)- and (*S*)-4-Methoxybenzyl [(4-methylcyclohex-3-en-1-yl) methyl]-carbamate (**19**)

To a solution of **18** (1.3 g, 9.3 mmol) in anhydrous toluene (20 mL) at 0 °C, diphenylphosphoryl azide (2.2 mL, 10.2 mmol) and Et_3_N (3.9 mL, 27.8 mmol) were added. The resulting mixture was left stirring for 3 h at 100 °C before 4-methoxybenzyl alcohol (1.27 mL, 10.2 mmol) was added, and the reaction was left to stir for 16 h at 100 °C. The reaction was then allowed to cool to room temperature and the solution was concentrated under reduced pressure. The residue was the purified by flash chromatography on silica gel (EtOAc : *n*-hexane 1 : 9) to yield **19** as a yellow crystalline solid (1.28 g, 80%). *δ*_H_ (300 MHz, CDCl_3_) *δ* 7.23 (2 H, dt, *J* = 2.9 and 5.3 C*H* ArCH), 6.81 (2 H, dt, *J* = 2.9 and 5.3, ArCH), 5.21 (1 H, br, *H*C=C), 4.95 (2 H, s, –OC*H*_2_Ph), 4.67–460 (1 H, m, CHN), 3.74 (3 H, s, –OCH_3_), 2.29–2.20 (2 H, m, CH_2_–C*H*_2_CHN), 1.93 (2 H, m, C*H*_2_–CH_2_CHN), 2.01–1.86 (2 H, m, CH–C*H*_2_CHN), 1.55 (3 H, s, CH_3_C=CH). *δ*_C_ (75 MHz, CDCl_3_) *δ* 159.5 (= *C*O–CH_3_), 155.8 (NH*C*=O), 134.1 (C= *C*CH_3_), 130.0 (*C*=C aromatic), 128.7 (*C*CH_2_O), 118.3 (*C*=CCH_3_), 113.9 (*C*=C aromatic), 66.3 (C*C*H_2_O), 62.8 (*C*HN), 55.3 (O*C*H_3_), 31.9 (*C*H_2_CHN), 28.4 (*C*H_2_C=C), 28.0 (*C*H_2_C=C), 23.4 (=C*C*H_3_). *ν*_max_ (thin film, cm^–1^) 3300 (N–H stretch), 2900–2700 (C–H stretch), 1650 (C=O ester stretch), 1250 (C–N stretch), 830 (aromatic CH bending); (*S*)-**19**: *α*_D_ –9.3, (*c* = 0.6, CHCl_3_) (*R*)-**19**: *α*_D_ +12 (*c* = 0.6, CHCl_3_) m.p. 69–71 °C LRMS (EI^+^) *m*/*z*: 275.15 (100% M^+^), 276.15 (20), 259.12 (18), 258.12 (60), 231.12 (25), 228.1128(12), 214.16 (100). HRMS (EI^+^) 275.1522; C_16_H_21_NO_3_ requires 275.1521.

### (*R*)- & (*S*)-*N*-Methyl-1-(4-methylcyclohex-3-en-1-yl)methanamine (**20**)

To a stirred solution of carbamate **19** (100 mg, 0.4 mmol) in anhydrous diethyl ether (7 mL) at 0 °C, was added LiAlH_4_ (50 mg, 1.28 mmol). The mixture was then heated to reflux for 5 h. The reaction was cooled to 0 °C before it was quenched by the addition of water (6 mL) and an excess of 15% NaOH solution (6 mL). The resulting mixture was left to stir at 0 °C for 1 h and the precipitate was removed by filtration through a Celite pad. The organic phase was extracted with water (2 × 10 mL) and the pooled organic layers were then washed with 10% HCl (2 × 10 mL) and the organic fraction was discarded. The combined aqueous layers were adjusted to pH 12 by dropwise addition of 10% NaOH (15 mL). The product was extracted with diethyl ether (4 × 15 mL), then dried over anhydrous MgSO_4_, and filtered. The product was then concentrated carefully under reduced pressure to give **20** as a volatile colorless oil (20 mg, 40%). *δ*_H_ (300 MHz, CDCl_3_) 5.24 (1 H, m br, C*H*=C), 2.55 (1 H, dt, *J* = 16.6 and 8.0, C*H*NH_2_), 2.37 (3 H, s, HNC*H*_3_), 2.25–2.10 (1 H, m, N*H*), 1.99–1.87 (2 H, m, C*H*_2_CH=C), 1.87–1.66 (2 H, m, CH_2_C*H*_2_C=CH), 1.61 (1 H, broad m, C*H*_2_CH_2_C=CH), 1.60 (3 H, s, H_2_C=CC*H*_3_), 1.45–1.26 (1 H, m, C*H*_2_CH_2_C=CH). *δ*_C_ (75 MHz, CDCl_3_) 134.0 (C= *C*CH_3_), 119.1 (*C*=CCH_3_), 54.8 (*C*HNH), 32.7 (*C*H_2_CHNH), 32.1 (C=CH*C*H_2_CH), 29.7 (*C*H_3_N), 29.0 (*C*H_2_C=), 23.4 (*C*H_3_C=) (*S*)-**20**: *α*_D_ –79 (*c* 1.00, CHCl_3_) (*R*)-**20**: *α*_D_ +84 (*c* 1.00, CHCl_3_) Data in agreement with previous work.14*a*

### (*R*)- and (*S*)-*N*,4-Dimethyl-*N*-(4-methylcyclohex-3-en-1-yl)pent-3-enamide (**21**)

To a stirred solution of **20** (172 mg, 1.5 mmol) and DIPEA (775 mg, 6.0 mmol) in anhydrous DMF (6 mL), HBTU (1.15 g, 3.0 mmol) was added and the resulting mixture was stirred at room temperature for 20 min before **20** (358 mg, 1.5 mmol) was added. The reaction was then stirred for 24 h at room temperature. The mixture was concentrated under reduced pressure and the residue was dissolved in diethyl ether (20 mL). The solution was washed with water (2 × 25 mL), 10% NaHCO_3_ (2 × 25 mL), 10% HCl (2 × 10 mL), and brine (25 mL) before it was dried over anhydrous MgSO_4_ and concentrated under reduced pressure. The residue was purified by flash chromatography on silica gel (EtOAc : hexane 4 : 6) yielding **21** as a colorless oil (166 mg, 50%). *δ*_H_ (400 MHz, CDCl_3_) 5.24 (2 H, dd, *J* = 14.6 and 8.0, NCHCH_2_C*H*= and =C*H*CH_2_C=O), 4.70–4.54 (0.5 H, m, C*H*N), 3.75 (0.5 H, dd, *J* = 12.6 and 9.7, C*H*N), 3.01 (2 H, dd, *J* = 12.6, 6.8 Hz, C*H*_2_C=O), 2.75 (1.5 H, s, C*H*_3_N), 2.72 (1.5 H, s, C*H*_3_N), 2.20–1.86 (5 H, m, C*H*_2_C*H*_2_CHC*H*_2_), 1.71–1.88 (1 H, m, CH_2_CH_2_CHC*H*_2_), 1.75 (s, 3 H, CNCH_2_=CHC*H*_3_), 1.68 (3 H, s, =CC*H*_3_), 1.66 (3 H, s, =CC*H*_3_). (*S*)-**21**: *α*_D_ –9.5, (CHCl_3_, *c* = 0.9) (*R*)-**21**: *α*_D_ +10, (CHCl_3_, *c* = 0.9) HRMS (EI^+^) 221.1779; C_14_H_23_NO requires 221.1780. Data are in agreement with previous work.14*a*

### (*R*)- and (*S*)-*N*,4-Dimethyl-*N*-(4-methylpent-3-en-3-yl)cyclohex-3-en-1 ammonium chloride (**11**)

To a stirred solution of **21** (41 mg, 0.19 mmol) in anhydrous diethyl ether at 0 °C, was added LiAlH_4_ (33 mg, 0.87 mmol). The mixture was heated to reflux for 6 h then allowed to cool to room temperature and stirred for a further 12 h. The reaction was quenched by the addition of water (6 mL) and 15% NaOH (6 mL) at 0 °C and stirred for 1 h at 0 °C. The white precipitate was removed by filtration on Celite and the filtrate was extracted with diethyl ether (2 × 25 mL). The combined organic layers were dried over anhydrous Na_2_SO_4_, concentrated under reduced pressure, and the residue was purified by flash chromatography on silica (Et_2_O : MeOH 1 : 9) to yield the amine as a yellow oil (32 mg, 65% yield). *δ*_H_ (300 MHz, CDCl_3_) 5.27 (1 H, d, *J* = 2.4, H_3_CC=C*H*), 5.03 (1H, t, *J* = 5.6, (CH_3_)_2_C=C*H*), 2.64–2.44 (1 H, m, C*H*N), 2.38 (2 H, ddd, *J* = 7.6, 5.8 and 2.6, C*H*_2_N), 2.23 (3 H, s, C*H*_3_N), 2.08 (2 H, dd, *J* = 15.5 and 7.2, C*H*_2_CH_2_N), 2.04–1.85 (4 H, m, C*H*_2_C*H*_2_CHN), 1.85–1.67 (2 H, m, C=CHC*H*_2_CHN), 1.62 (3 H, s, C*H*_3_C=CH), 1.55 and 1.57 (2 × 3 H, 2 × s, (C*H*_3_)_2_C=CH). *δ*_C_ (75 MHz, CDCl_3_) *δ* 133.9 (HC= *C*CH_3_), 132.6 (CH= *C*CH_3_), 122.1 (*C*H=CCH_3_), 120.0 (NCH_2_CH_2_*C*H=CCH_3_), 58.9 (*C*HN), 53.5 (*C*H_2_N), 37.9 (CH_2_*C*H_2_CHN), 30.8 (=CH*C*H_2_N), 27.2 (*C*H_2_CCH_3_), 26.5 (CH_3_N), 25.7(*C*H_2_CH_2_N), 25.6 (*C*H_3_CCH_3_), 23.2 (CH_3_C*C*H_3_), 17.8 (*C*H_3_C=). HRMS (EI^+^) 207.1990; C_14_H_25_N requires 207.1987. (*S*)-**11**: *α*_D_ –61 (CHCl_3_, *c* = 1) (*R*)-**11***α*_D_ +63 (CHCl_3_, *c* = 1). Data are in agreement with previous work.14*a* The amine was then dissolved in Et_2_O (1 mL) and HCl (1 M in anhydrous Et_2_O) was added slowly. A light yellow precipitate was formed. The ether was concentrated under reduced pressure and the salt stored in 1.2 mL of deionised water. *δ*_H_ (300 MHz, MeOD) 5.38 (1 H, br, H_3_CC=C*H*), 5.14 (1 H, t, *J* = 5.0, (CH_3_)_2_C=C*H*), 3.58–3.42 (1 H, m, C*H*N), 3.32 (1 H, dd, *J* = 4.9, 1.6, C*H*HCH_2_N), 3.25–2.97 (1 H, m, CH*H*CH_2_N), 2.85 (3 H, s, C*H*_3_N), 2.59–2.39 (2 H, m, C*H*_2_CH_2_CHN), 2.39–2.26 (2 H, m, CH_2_C*H*_2_CHN), 2.26–2.05 (2 H, m, C=CHCH_2_C*H*_2_N), 1.84 (2 H, dd, *J* = 12.2, 10.4 Hz, CH_3_C=CHC*H*_2_), 1.76 (3 H, s, C*H*_3_C=CH), 1.72 (6 H, 2 × s, (CH_3_)_2_C=CH). *δ*_C_ (75 MHz, MeOD) *δ* 136.1 (HC= *C*CH_3_), 134.3 (CH= *C*(CH_3_)_2_) 117.6 (*C*H=CCH_3_) 116.5 (NCH_2_CH_2_*C*H=CCH_3_), 61.8, 61.5 (*C*HN), 53.0, 52.5 (*C*H_2_N), 35.9, 35.2 (CH_3_N) 29.1, 29.0 (CH_2_*C*H_2_CHN), 25.7 (=CH*C*H_2_N), 24.5 (*C*H_2_CCH_3_), 24.2, 24.0 (NCH_2_*C*H_2_), 23.25, 22.64 (*C*H_3_C=), 21.64 (*C*H_3_CCH_3_), 16.65 (CH_3_C*C*H_3_). *ν*_max_ (neat, cm^–1^) 2972 (broad, N–H stretch), 1379 (C–N stretch), 1161, 1051 and 1022 (C–N stretch), 950, 879, 815; HRMS (APCI^+^) 208.2057, C_14_H_26_N requires 208.2065; m.p. 131–133 °C, (*S*)-**11***α*_D_ –62.2 (MeOH, *c* = 0.09), (*R*)-**11***α*_D_ +57.1 (MeOH, *c* = 0.09). Data are in agreement with previous work.14*a*

## Conflicts of interest

There are on conflicts of interest to declare.

## Supplementary Material

Supplementary information

## References

[cit1] (a) Connolly J. D. and Hill R. A., Dictionary of Terpenoids, Chapman and Hall, London, 1991.

[cit2] Cane D. (1990). Chem. Rev..

[cit3] Felicetti B., Cane D. E. (2004). J. Am. Chem. Soc..

[cit4] Degenhardt J., Kollner T. G., Gershenzon J. (2009). Phytochemistry.

[cit5] Jiang J. Y., He X. F., Cane D. E. (2007). Nat. Chem. Biol..

[cit6] Li J.-X., Fang X., Zhao Q., Ruan J.-X., Yang C.-Q., Wang L. J., Miller D. J., Faraldos J. A., Allemann R. K., Chen X. Y., Zhang P. (2013). Biochem. J..

[cit7] Faraldos J. A., Grundy D. J., Cascon O., Leoni S., van der Kamp M. W., Allemann R. K. (2017). Chem. Commun..

[cit8] Steele C. L., Crock J., Bohlmann J., Croteau R. (1998). J. Biol. Chem..

[cit9] Davis G. D., Essenberg M. (1995). Phytochemistry.

[cit10] Benedict C. R., Lu J. L., Pettigrew D. W., Liu J., Stipanovic R. D., Williams H. J. (2001). Plant Physiol..

[cit11] Bianchini G. M., Stipanovic R. D., Bell A. A. (1999). J. Agric. Food Chem..

[cit12] Faraldos J. A., Miller D. J., González V., Yoosuf-Aly Z., Cascón O., Li A., Allemann R. K. (2012). J. Am. Chem. Soc..

[cit13] Faraldos J. A., Kariuki B. M., Coates R. M. (2011). Org. Lett..

[cit14] Cane D. E., Yang G., Coates R. M. (1992). J. Org. Chem..

[cit15] Poll T., Sobczak A., Hartmann H., Helmchen G. (1985). Tetrahedron Lett..

[cit16] (b) Watkins W. J., Preparation of thiophencarboxylic acid derivatives for use as flaviviridae viruses inhibitors, US Patent 20130052161, 2013.

[cit17] Calvert M., Taylor S. E., Allemann R. K. (2002). Chem. Commun..

[cit18] Demiray M., Tang X., Faraldos J. A., Wirth T., Allemann R. K. (2017). Angew. Chem., Int. Ed..

[cit19] Loizzi M., González V., Miller D. J., Allemann R. K. (2018). ChemBioChem.

[cit20] Tantillo D. J. (2017). Angew. Chem., Int. Ed..

[cit21] Tang X., Demiray M., Wirth T., Allemann R. K. (2017). Bioorg. Med. Chem..

[cit22] Miller D. J., Yu F., Allemann R. K. (2007). ChemBioChem.

[cit23] Touchet S., Chamberlain K., Woodcock C. M., Miller D. J., Birkett M. A., Pickett J. A., Allemann R. K. (2015). J. Am. Chem. Soc..

[cit24] Huynh F., Miller D. J., Allemann R. K. (2018). ChemBioChem.

