# Supplementary material for: Silent catalytic promiscuity in the high-fidelity terpene cyclase δ-cadinene synthase
Source: Org Biomol Chem. 2019 Jan 17;17(5):1206–14. doi: 10.1039/c8ob02821d (PMC6369673; doi:10.1039/c8ob02821d)
Supplement: Supplementary file 1 [file OB-017-C8OB02821D-s001.pdf]

Electronic Supplementary Material (ESI) for Organic & Biomolecular Chemistry.
This journal is © The Royal Society of Chemistry 2018

Electronic Supplementary information for:

Silent catalytic promiscuity in the high-fidelity terpene cyclase δ-cadinine synthase

Marianna Loizzi,[a] David J. Miller[a] and Rudolf K. Allemann[a*]

[a] School of Chemistry, Cardiff University, Main Building, Park Place, Cardiff. CF10 3AT

Table of Contents

1. **Materials and general methods** 2
2. **Steady-state kinetics of DCS-His$_6$, ADS and DCS** 3
3. **Kinetic assay results** 4
4. **GC-MS results** 9
5. **Chiral HPLC results** 12
6. **NMR spectra** 14
7. **References** 21

1. Materials and general methods. δ–cadinene synthase (DCS) from *Gossypium arboreum* was produced as previously described[1] using competent cells from *E.coli* BL-21 (DE3). Amorphadiene synthase (ADS) from *Artemisia annua* was produced as previously described by Demiray *et al*.[2] Aristolochene synthase (AS) from *Penicillum roqueforti* was produced as previously described in the literature.[3] Protein expression was induced by the use of isopropyl-thio-β-D-galactopyranoside (IPTG). Nickel affinity chromatography was used for protein purification of DCS and ADS. A diethylaminoethyl anion exchange column (DEAE, 75 mL) was used for AS purification. A prestained protein size marker (6.5-175kD) was used to identify protein by 10% SDS gel. An Amicon YM30 membrane was used for protein concentration. [1-^{3}H-FDP] (20Ci mmol) was purchased by American Radiolabeled Chemicals. Unlabelled FDP was available from previous studies and synthesised following Davisson's two steps protocol (chlorination/diphosphorylation) with modification from commercially available (*E*,*E*)-farnesol.[4] Commercial [1-^{3}H]-FDP was diluted by adding cold FDP to give a final specific activity of 50 mCi/mmol.

For synthetic procedures, all chemicals and solvents were obtained from commercial vendors and used without further purification unless otherwise noted. Anhydrous tetrahydrofuran (THF), diethyl ether, toluene and acetonitrile were obtained from a MBraun SPS800 solvent purification system. Dichloromethane (CH_2Cl_2) and trimethylamine (Et_3N) were distilled under nitrogen over calcium hydride and potassium hydroxide, respectively. ^{1}H NMR, and ^{13}C NMR, spectra were measured on Bruker Avance III 600, Bruker Avance 500, Bruker Avance III HD 400, and Bruker Fourier 300 NMR spectrometers. The spectra are reported in order as chemical shifts in parts per million (ppm), downfield from tetramethylsilane (^{1}H and ^{13}C), integral, multiplicity, coupling and assignment, respectively. Assignments are made to the limitations of COSY, DEPT 90/135, gradient HSQC, and gradient HMBC spectra. Mass spectra were measured on a Waters GCT Premier time of flight mass spectrometer and a Waters LCT time of flight mass spectrometer.

GC-MS analysis of incubation products was performed on a Waters GCT Premier apparatus fitted with: column A = J&W Scientific DB-5MS column (30 m x 0.25 mm internal diameter), column B= Agilent J&W DB-35MS (30 m x 0.25 mm internal diameter), and a Micromass GCT Premiere detecting in the range m/z 50-800 in EI+ mode with a scan every 0.95 s with a scan time of 0.9 s. Method 1: injection temperature 100 °C; with a split ratio of 5:1; initial pressure 1 kPa, scans in function: 1525 ; initial oven temperature 80 °C, ramp of 4 °C/min to 180 °C (2 min hold) , flow 1 mL/min, retention window 0 to 28 min; Method 2: injection port at 100 °C; split ratio 5:1; initial pressure 1 kPa; scans

in function: 1449; initial temperature 80 °C hold, ramp of 4 °C/min to 180 °C (15 min hold), then 20 °C/min to 250 °C, flow 1 mL/min, retention window 0 to 28 min.

Thin layer chromatography (TLC) was performed on pre-coated aluminium plates of silica G/UV254. TLC visualisations were performed with 4.2% ammonium molybdate and 0.2% ceric sulfate in 5% sulfuric acid (Hanessian's stain), or by UV light. The optical rotations were determinated using a UNiPol L-serie L1000/L2000 Schmidt+Haesch polarimeter. All measurements were performed at room temperature using a 5 cm long cell path and a wavelength of 269 nm.
Infrared (IR) spectra were obtained using a Jasco V-660 spectrophotometer.

Ion-exchange chromatography was performed using ion-exchange resin (Amberlyst 131 wet, H+ form) pre-equilibrated with ion-exchange buffer (25 mM NH_4HCO_3 containing 2% isopropanol, 2 CV). Reverse phase HPLC was performed on a system comprising of a Dionex P680 pump and a Dionex UVD170U detector unit. The column used was a 150 x 21.2 mm Phenomenex Luna C-18 column.

2. Steady-state kinetics of DCS-His$_6$, ADS and AS.

Kinetic assays were carried out according to the standard, linear range, micro-assay procedure previously described.[5] The reaction mixtures containing buffer, FDP and protein were prepared on ice in a total volume of 250 µL and were overlaid with ca. 0.8 mL of HPLC-grade hexane prior to incubation. The assay mixtures were initiated by addition of enzyme and incubated at room temp (30 °C) for 10 min. The reactions were immediately ice-cooled and quenched by addition of 200 µL of 100 mM EDTA (pH 8.5) and brief vortexing. The hexane overlay and two additional 1 mL hexane extracts were passed through a short pipette column containing silica gel. The column was washed with additional hexane (1 mL) and the combined filtrates were analysed by liquid scintillation counting using 15 mL of scintillation cocktail Ecoscint O. Steady-State kinetic parameters for DCS-His$_6$ and mutants were obtained by direct fitting of the data to the Michaelis-Menten equation by nonlinear least squares regression in conjunction with the graphical procedures developed by Lineweaver-Burk using the commercial SigmaPlot package (Systat Software).

3. Kinetic assay results

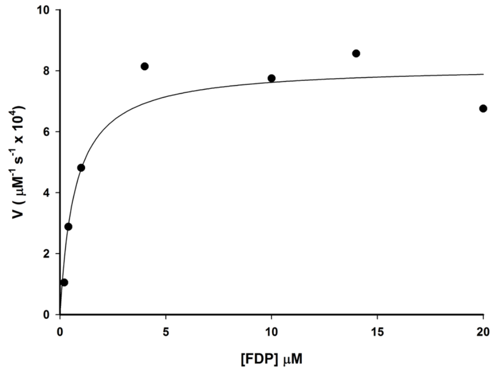

Figure S1. Representative Michaelis-Menten plot for the production of radiolabelled products by DCS-His_6.

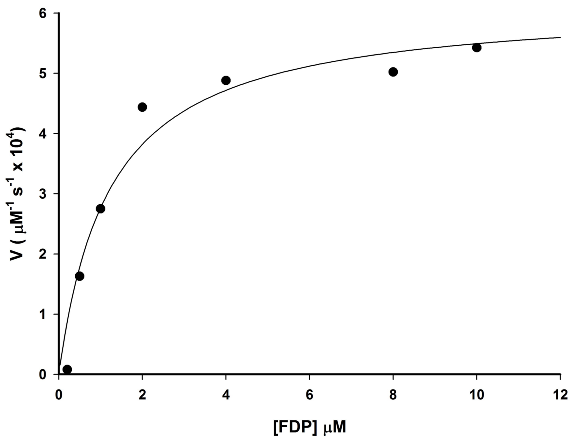

Figure S2. Representative Michaelis-Menten plots for the production of radiolabelled products by ADS.

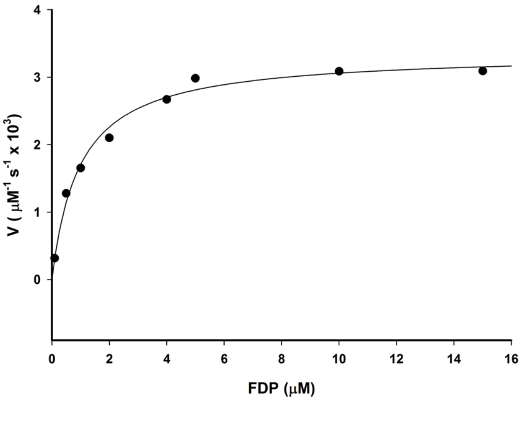

Figure S3. Representative Michaelis-Menten plots for the production of radiolabelled products by AS.

Representative Lineweaver-Burk plots

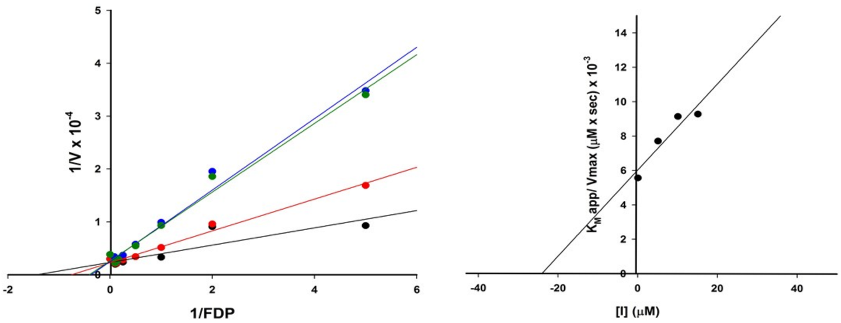

Figure S4. Left: Lineweaver-Burke plot for inhibition of ADS by (*S*)-**11**. Right: Plot of [(*S*)-**11**] versus $K_{M(app)}/V_{max}$ for determination of K_I.

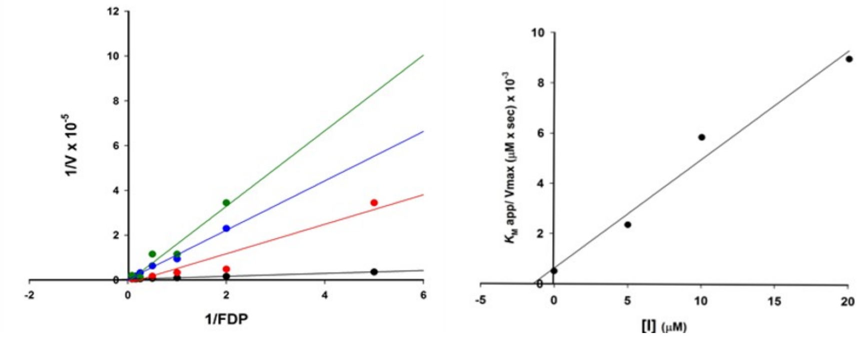

Figure S5. Left: Lineweaver-Burke plot for inhibition of ADS by (*S*)-11. Right: Plot of [(*S*)-11] versus $K_{M(app)}/V_{max}$ for determination of K_I.For each series of reaction a fixed concentration of 250 μM of PP_i was added.

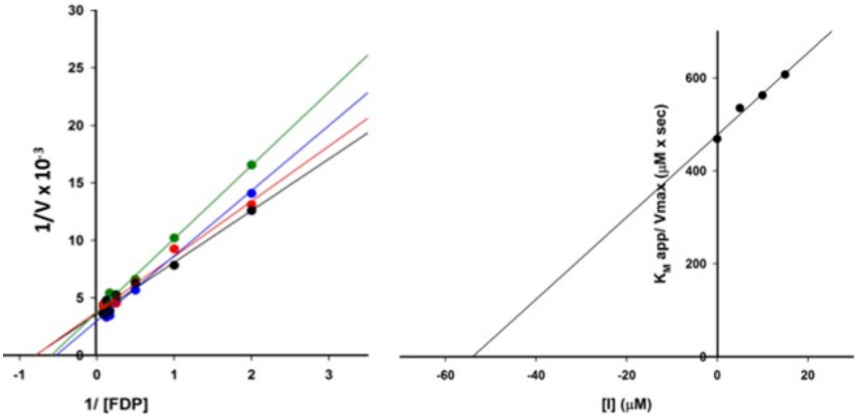

Figure S6. Left: Lineweaver-Burke plot for inhibition of ADS by (*R*)-**11**. Right: Plot of [(*R*)-**11**] versus $K_{M(app)}/V_{max}$ for determination of K_I.

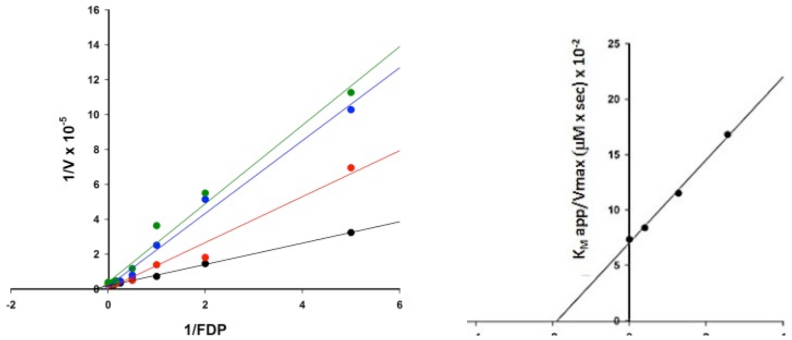

Figure S7. Left: Lineweaver-Burke plot for inhibition of ADS by (*S*)-**11**. Right: Plot of [(*S*)-**11**] versus $K_{M(app)}/V_{max}$ for determination of K_I. For each series of reactions a fixed concentration of 250 μM of PP_i was added.

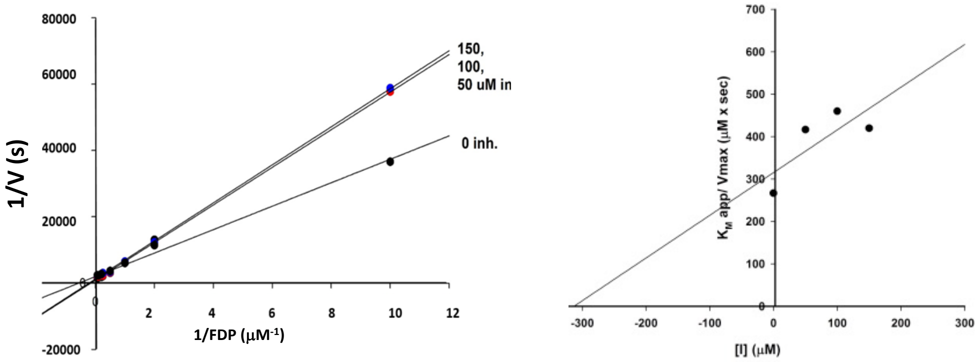

Figure S8. Left: Lineweaver-Burke plot for inhibition of AS by (*S*)-**11**. Right: Plot of [(*S*)-**11**] versus $K_{M(app)}/V_{max}$ for determination of K_I.

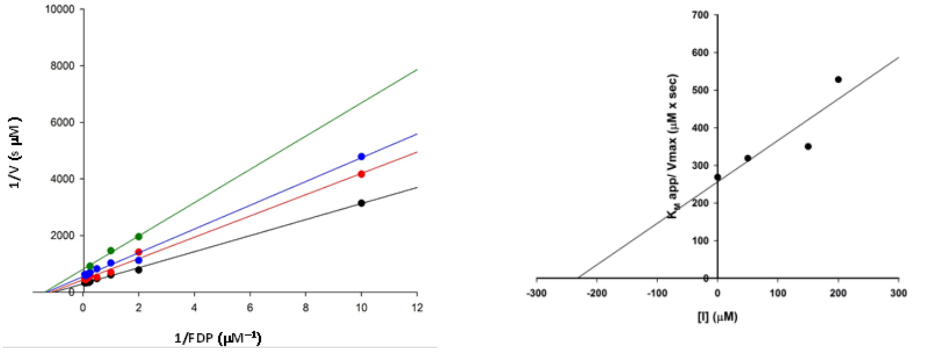

Figure S9. Left: Lineweaver-Burke plot for inhibition of AS by (*S*)-**11**. Right: Plot of [(*S*)-**11**] versus $K_{M(app)}/V_{max}$ for determination of K_I. For each series of reaction a fixed concentration of 250 μM of PP_i was added.

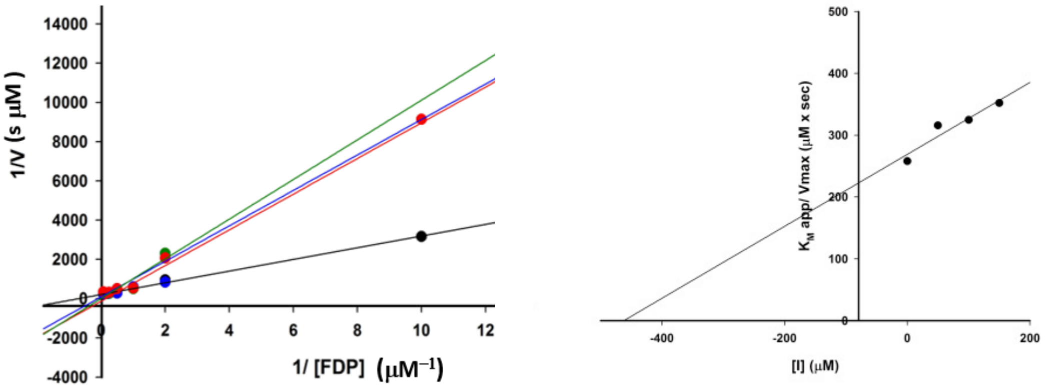

Figure S10. Left: Lineweaver-Burke plot for inhibition of AS by (*R*)-**11**. Right: Plot of [(*R*)-**11**] versus $K_{M(app)}/V_{max}$ for determination of K_I.

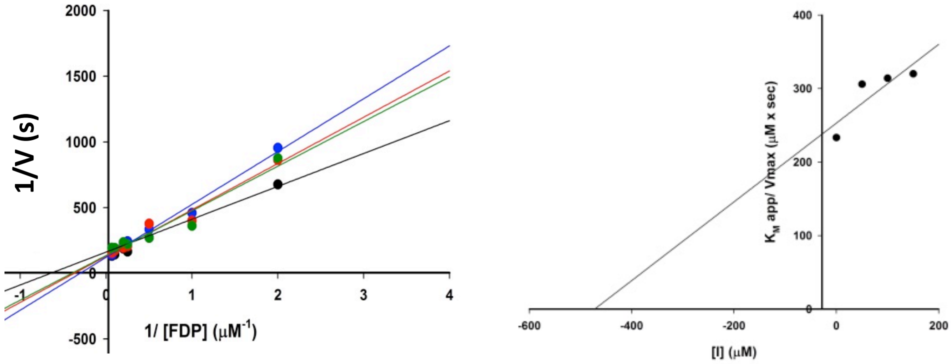

Figure S11. Left: Lineweaver-Burke plot for inhibition of AS by (*S*)-**11**. Right: Plot of [(*S*)-11] versus $K_{M(app)}/V_{max}$ for determination of K_I. For each series of reaction a fixed concentration of 250 μM of PP_i was added.

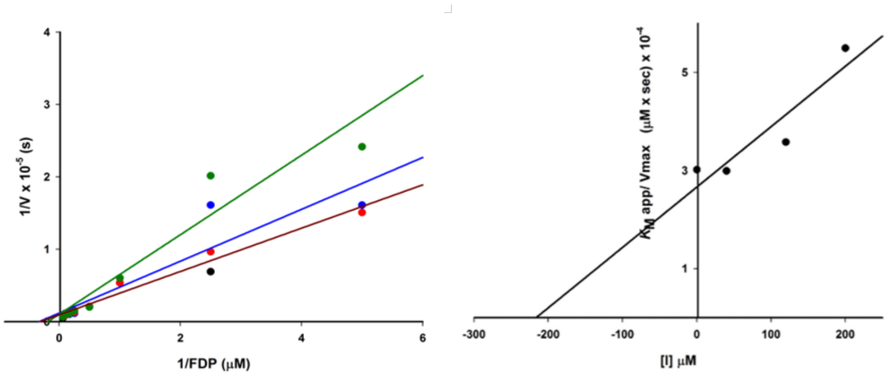

Figure S12. Left: Lineweaver-Burke plot for inhibition of DCS by (*S*)-**11**. Right: Plot of [(*S*)-**11**] versus $K_{M(app)}/V_{max}$ for determination of K_I.

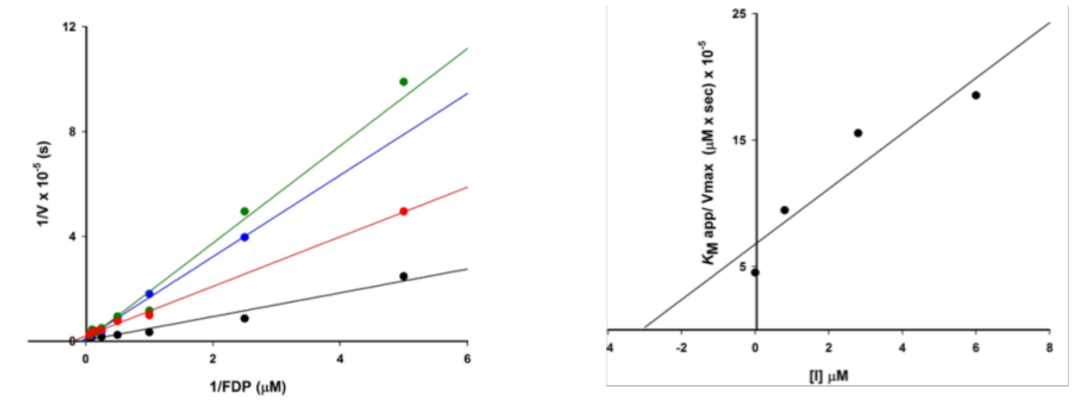

Figure S13. Left: Lineweaver-Burke plot for inhibition of DCS by (*S*)-**11**. Right: Plot of [(*S*)-**11**] versus $K_{M(app)}/V_{max}$ for determination of K_I. For each series of reaction a fixed concentration of 250 μM of PP_i was added.

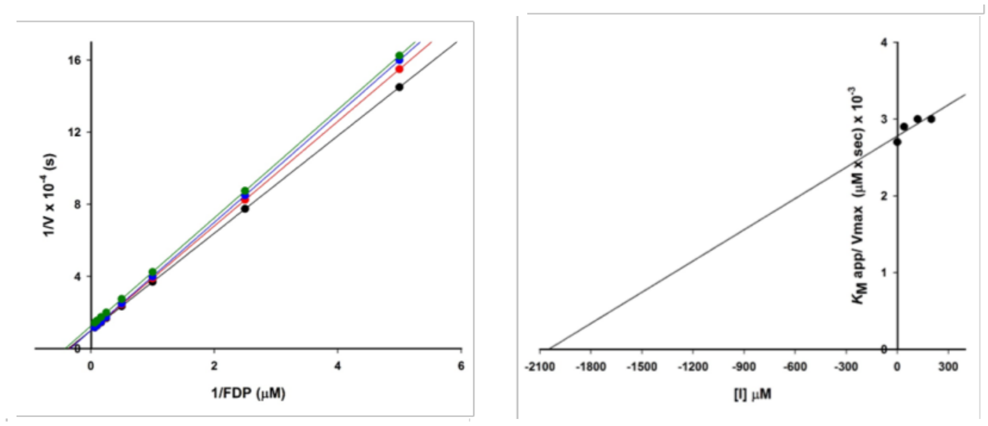

Figure S14. Left: Lineweaver-Burke plot for inhibition of DCS by (*R*)-**11**. Right: Plot of [(*R*)-**11**] versus $K_{M(app)}/V_{max}$ for determination of K_I.

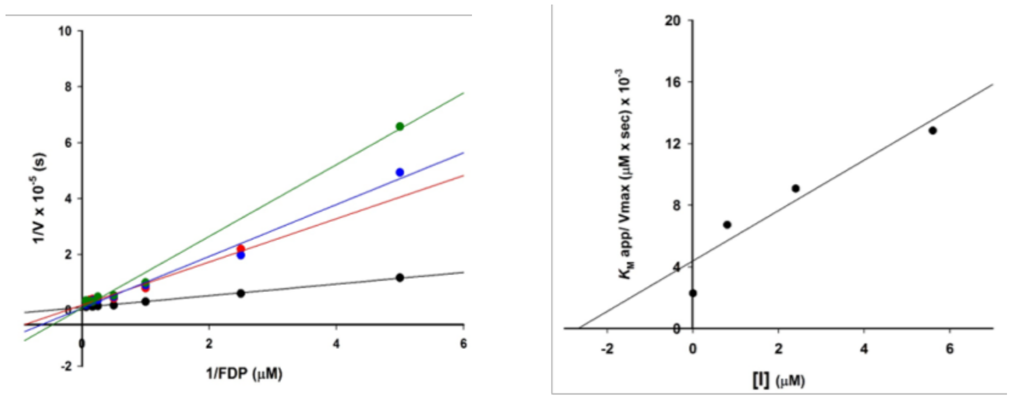

Figure S15. Left: Lineweaver-Burke plot for inhibition of DCS by (*R*)-**11**. Right: Plot of [(*R*)-**11**] versus $K_{M(app)}/V_{max}$ for determination of K_I. For each series of reactions a fixed concentration of 250 μM of PP_i was added.

4. GC-MS results

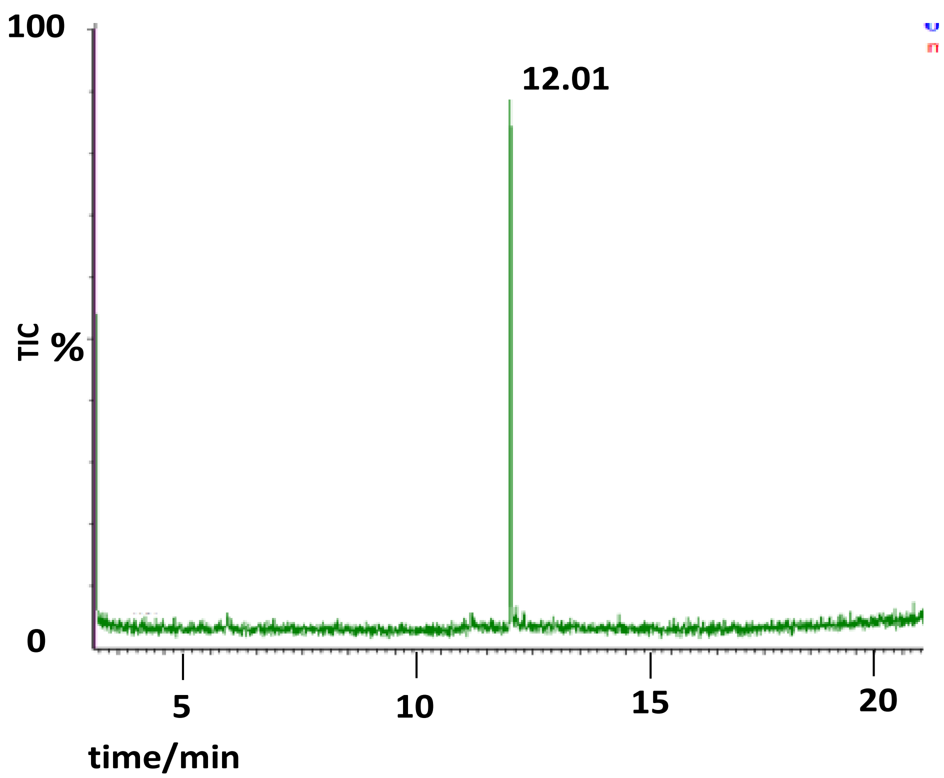

Figure S16. Total ion chromatogram of the pentane extractable products arising from incubation of AS with FDP.

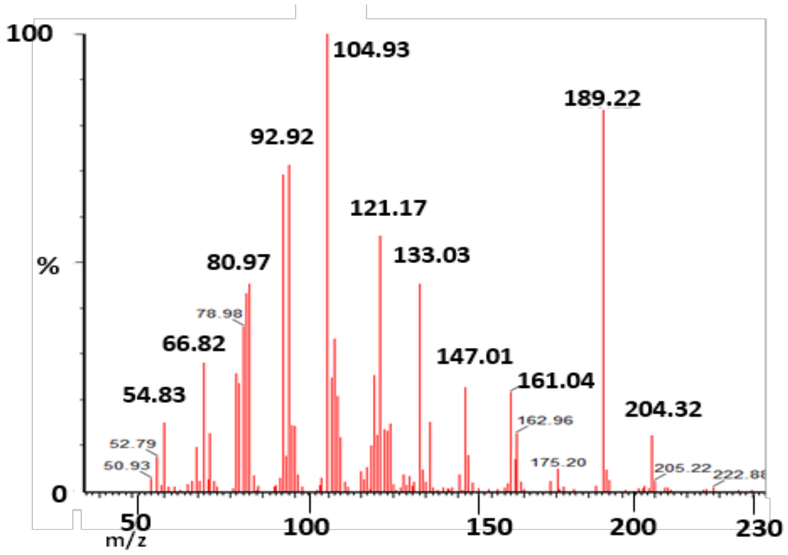

Figure S17. Mass spectrum of the compound eluting at 12.01 min: aristolochene.

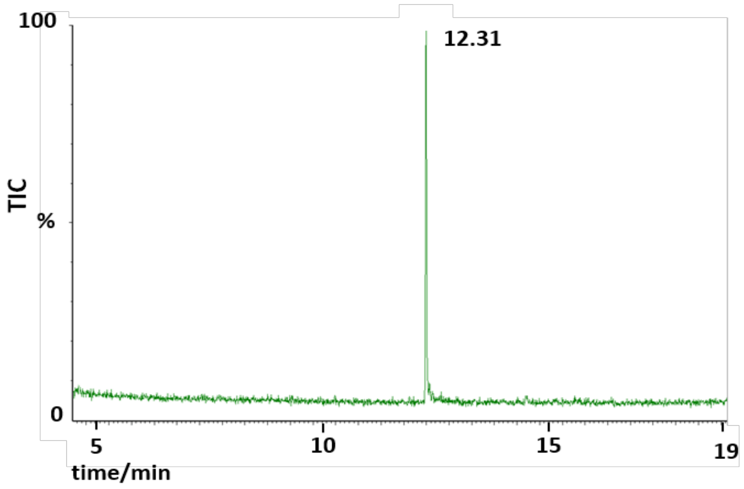

Figure S18. Total ion chromatogram of the pentane extractable products arising from incubation of ADS with FDP. Method 2, column B

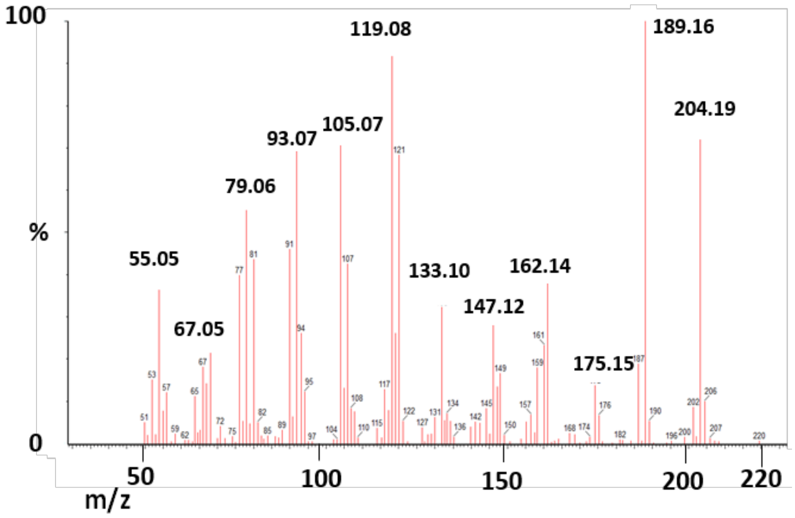

Figure S19. Mass spectrum of the compound eluting at 12.31 min: amorpha-4,11-diene.

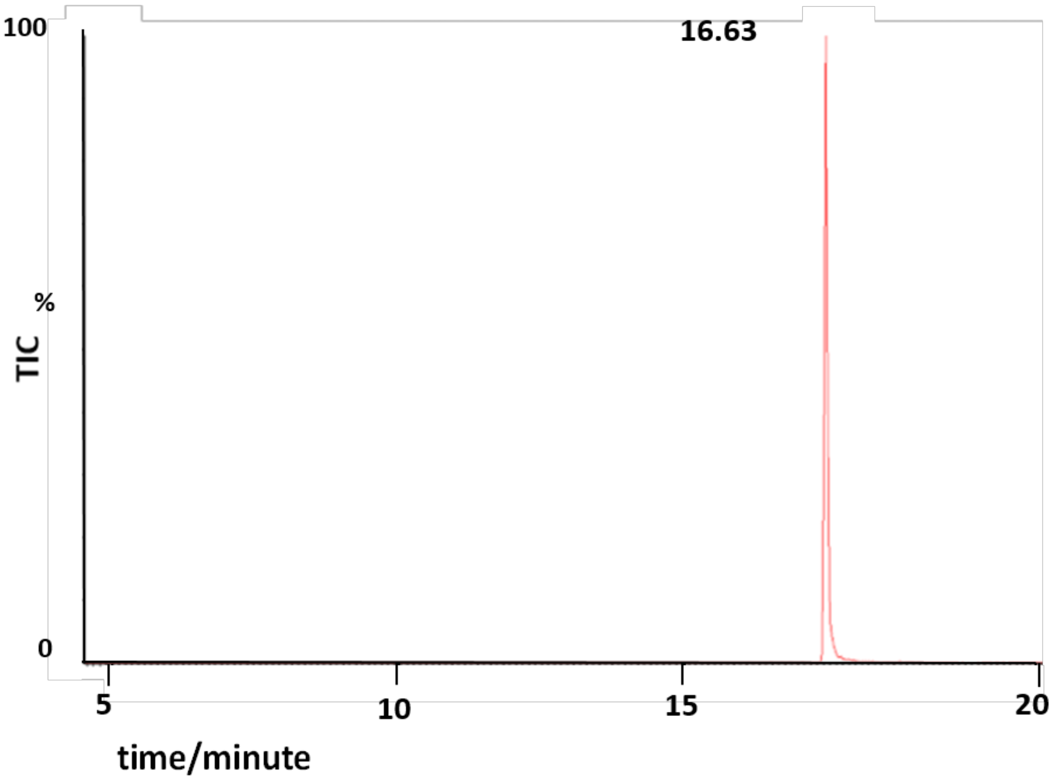

Figure S20. Total ion chromatogram of the pentane extractable products arising from incubation of DCS with FDP. Method 2, column B

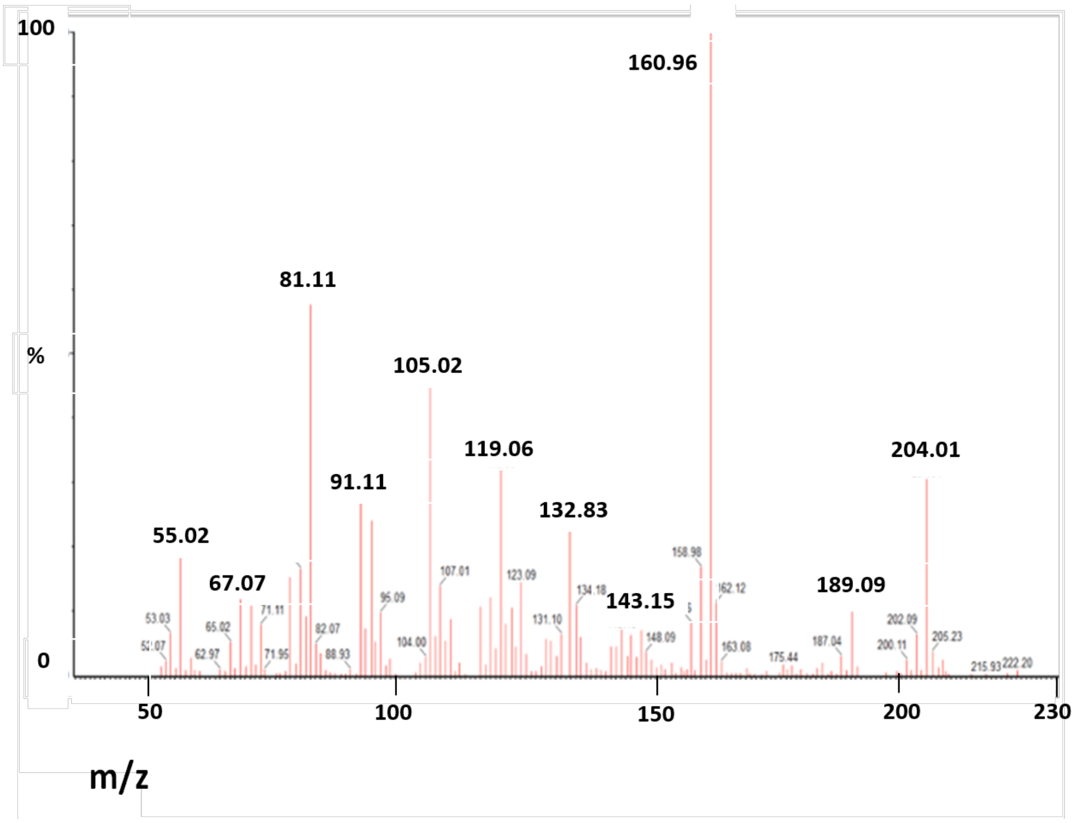

Figure S21. Mass spectrum of the compound eluting at 16.63 min: δ-cadinene.

5. Chiral HPLC results

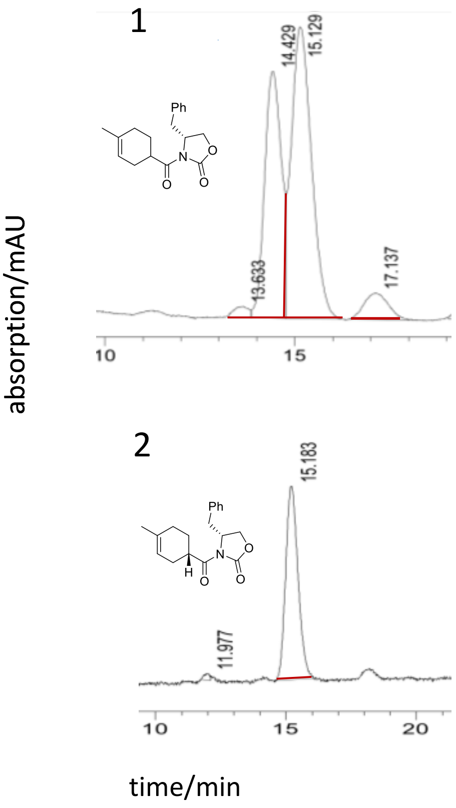

Figure S22. High performance liquid chromatogram of **14** over a chiral stationary phase. Top: catalyst $AlCl_3$. Bottom: catalyst DEAC**.**

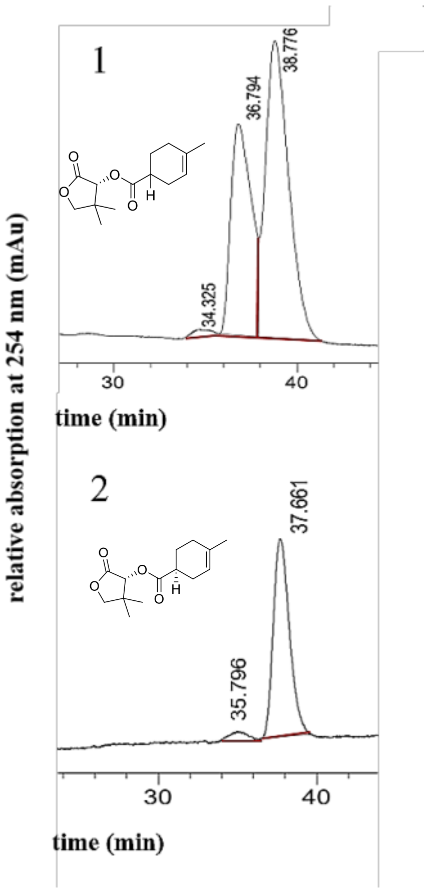

Figure S23. High performance liquid chromatograms of **17**. 1: racemic **17** and 2: **17** using a chiral stationary phase.

6. NMR spectra

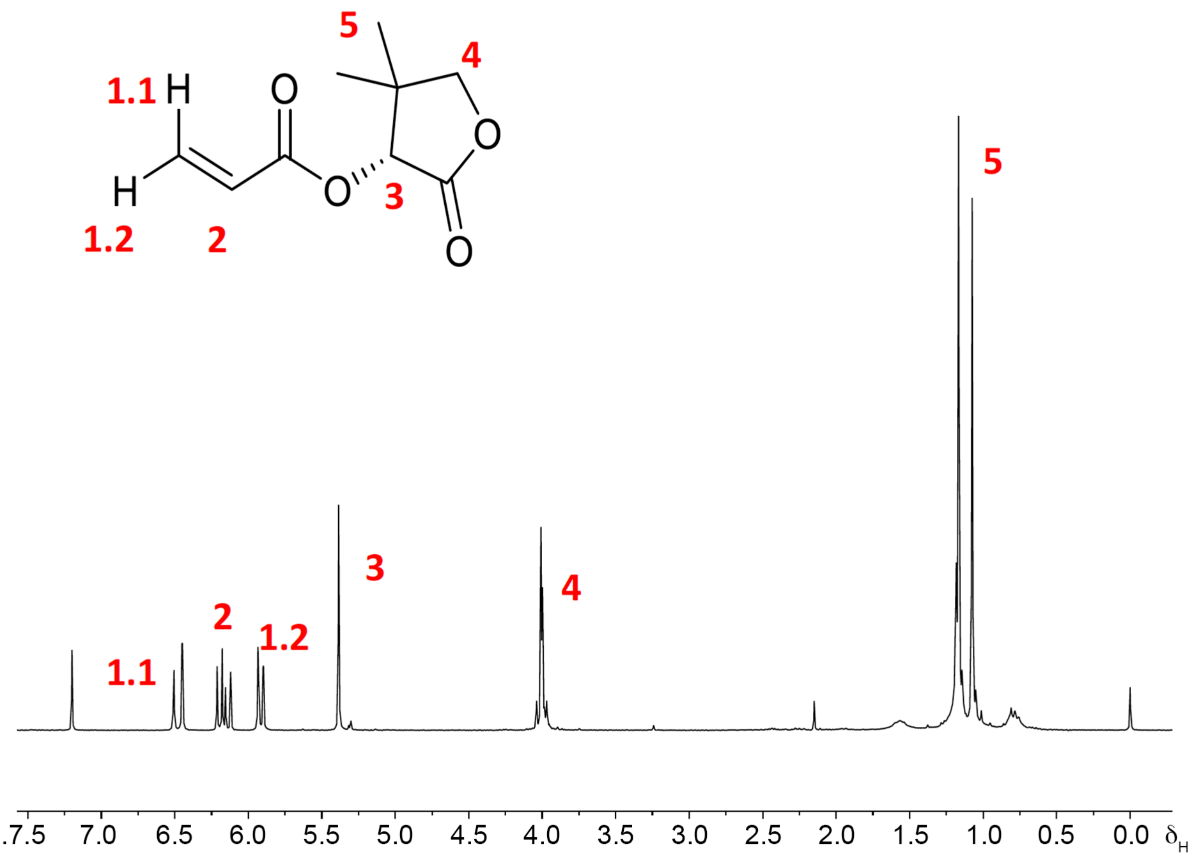

Figure S24. ^{1}H-NMR spectrum (300 MHz, $CDCl_3$) of **16**.

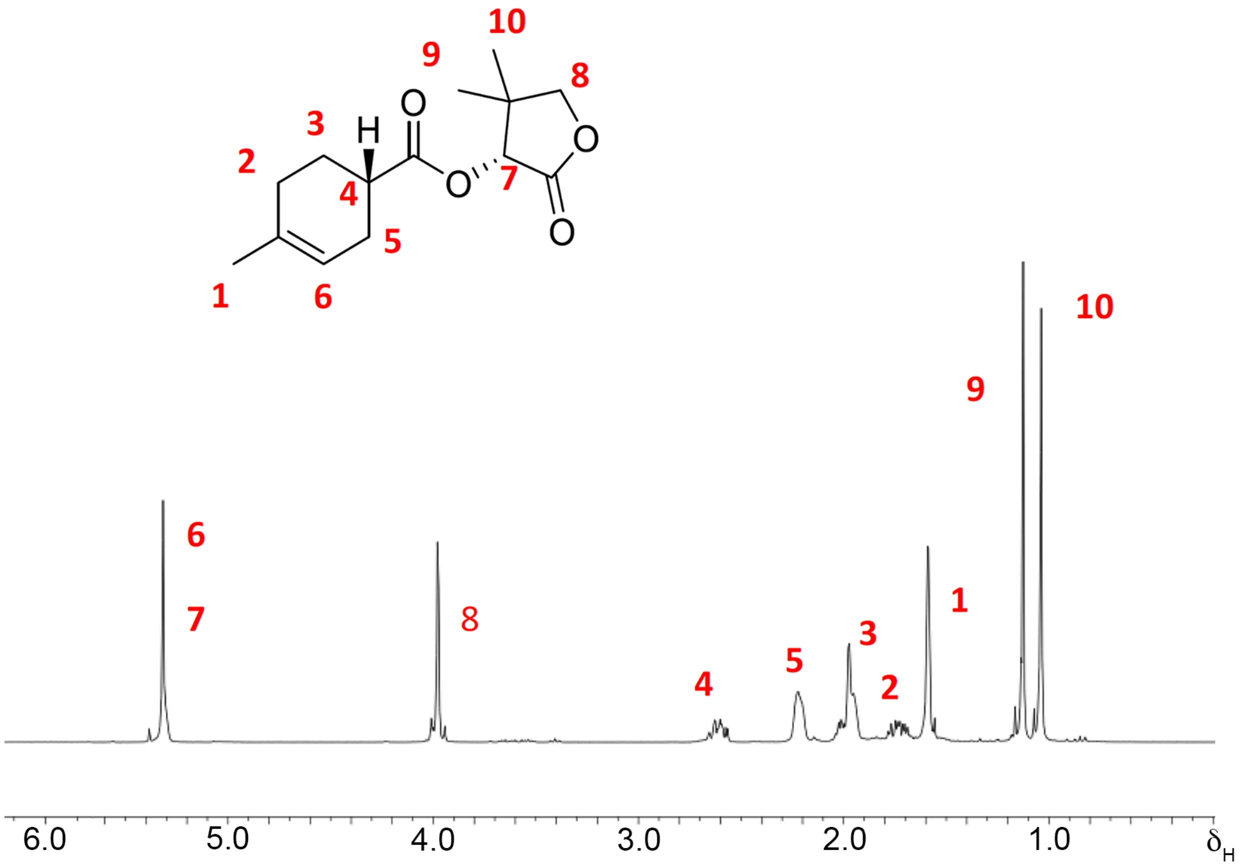

Figure S25. ^{1}H-NMR spectrum (300 MHz, $CDCl_3$) of **17**.

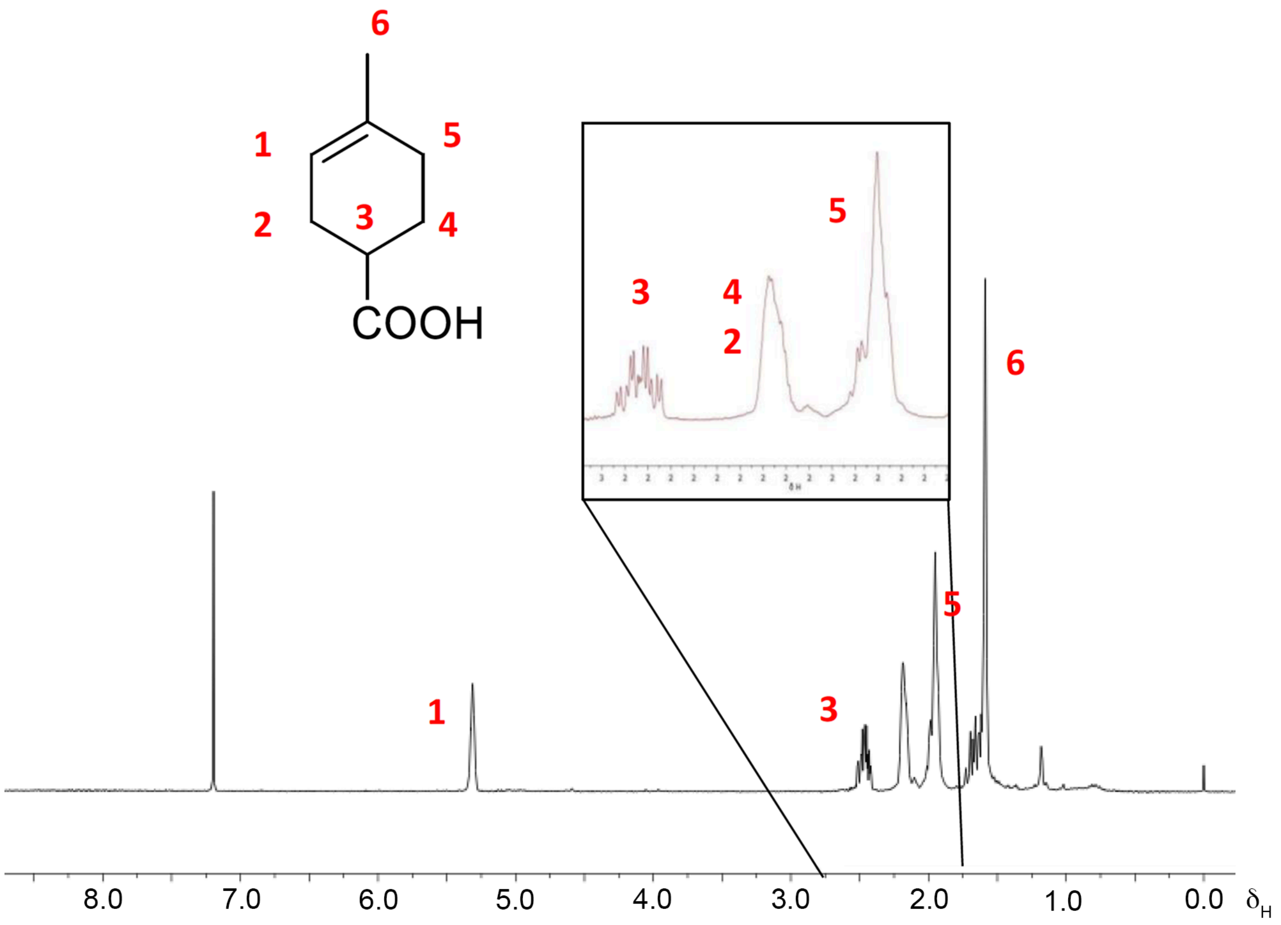

Figure S26. ^{1}H-NMR spectrum (300 MHz, $CDCl_3$) of **18**.

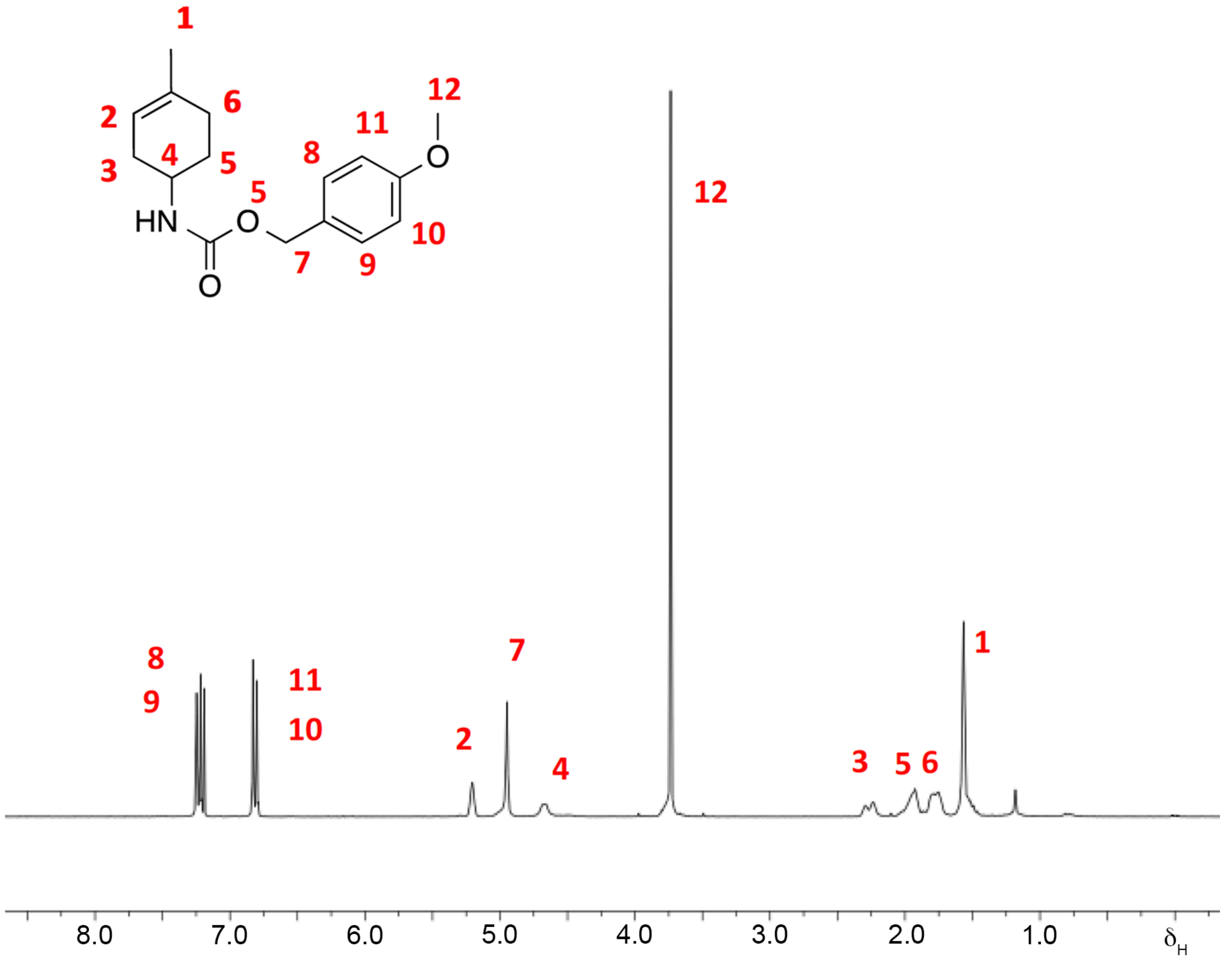

Figure S27. ^{1}H-NMR spectrum (300 MHz, $CDCl_3$) of **19**.

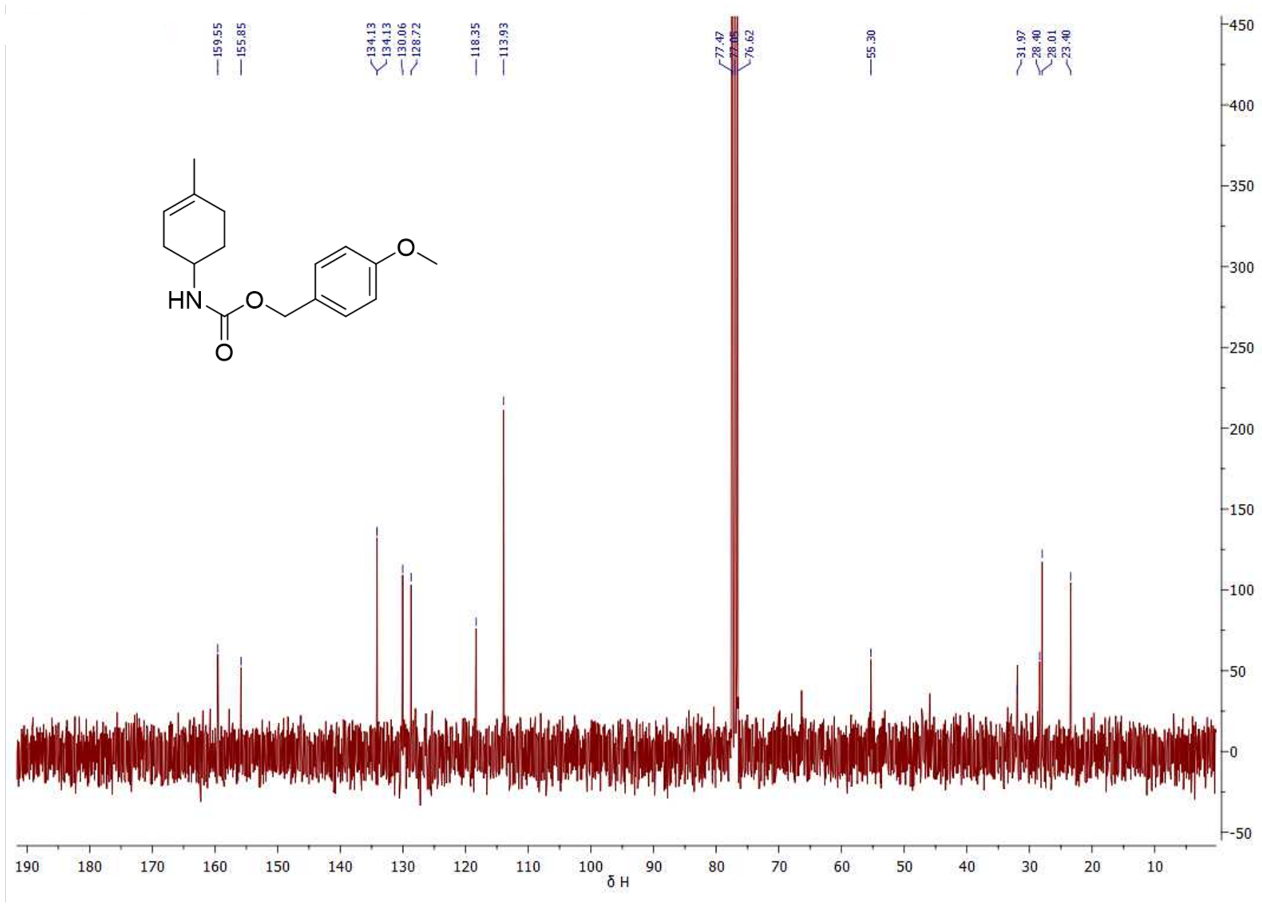

Figure S28. ^{13}C-NMR spectrum (75 MHz, $CDCl_3$) of **19**.

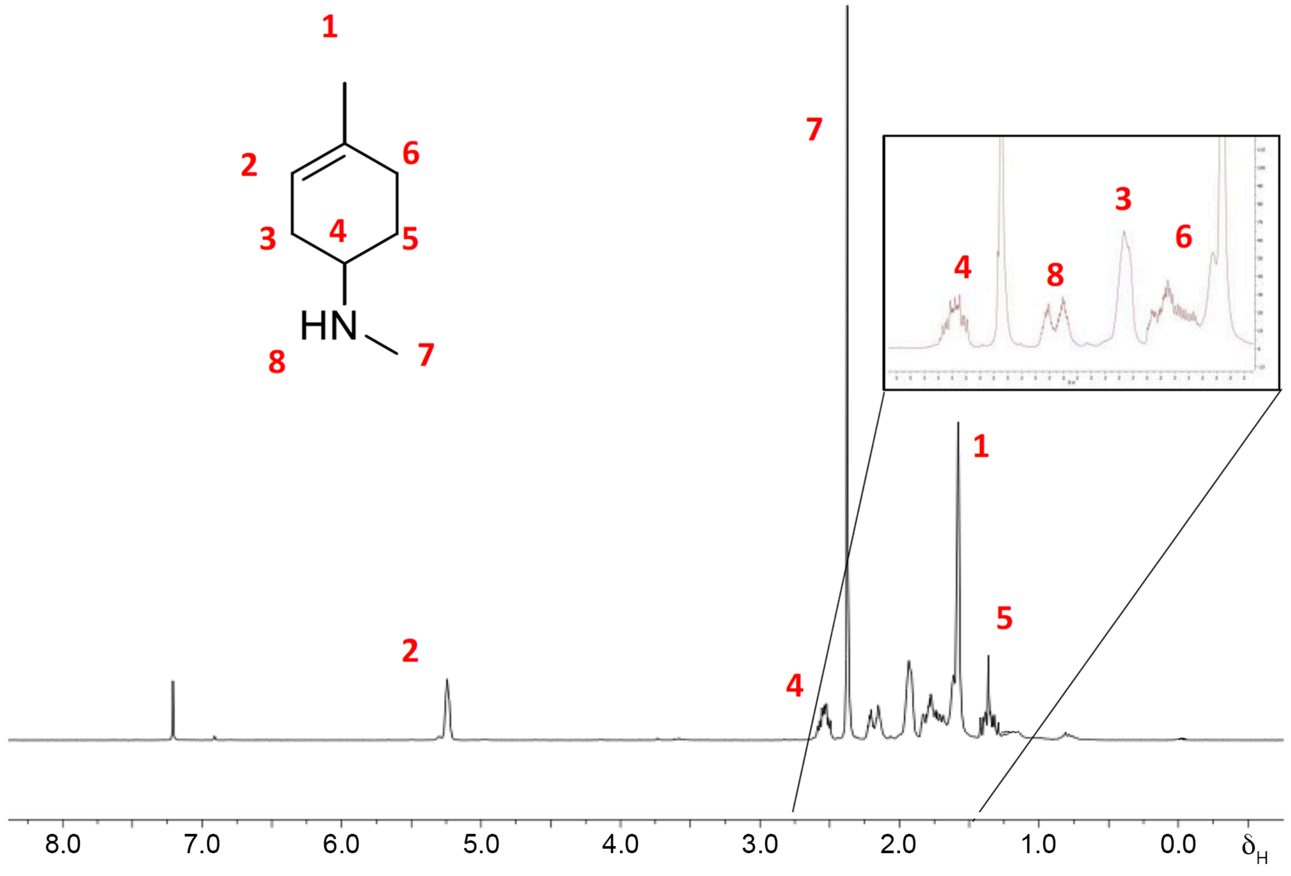

Figure S29. ^{1}H-NMR spectrum (300 MHz, $CDCl_3$) of **20**.

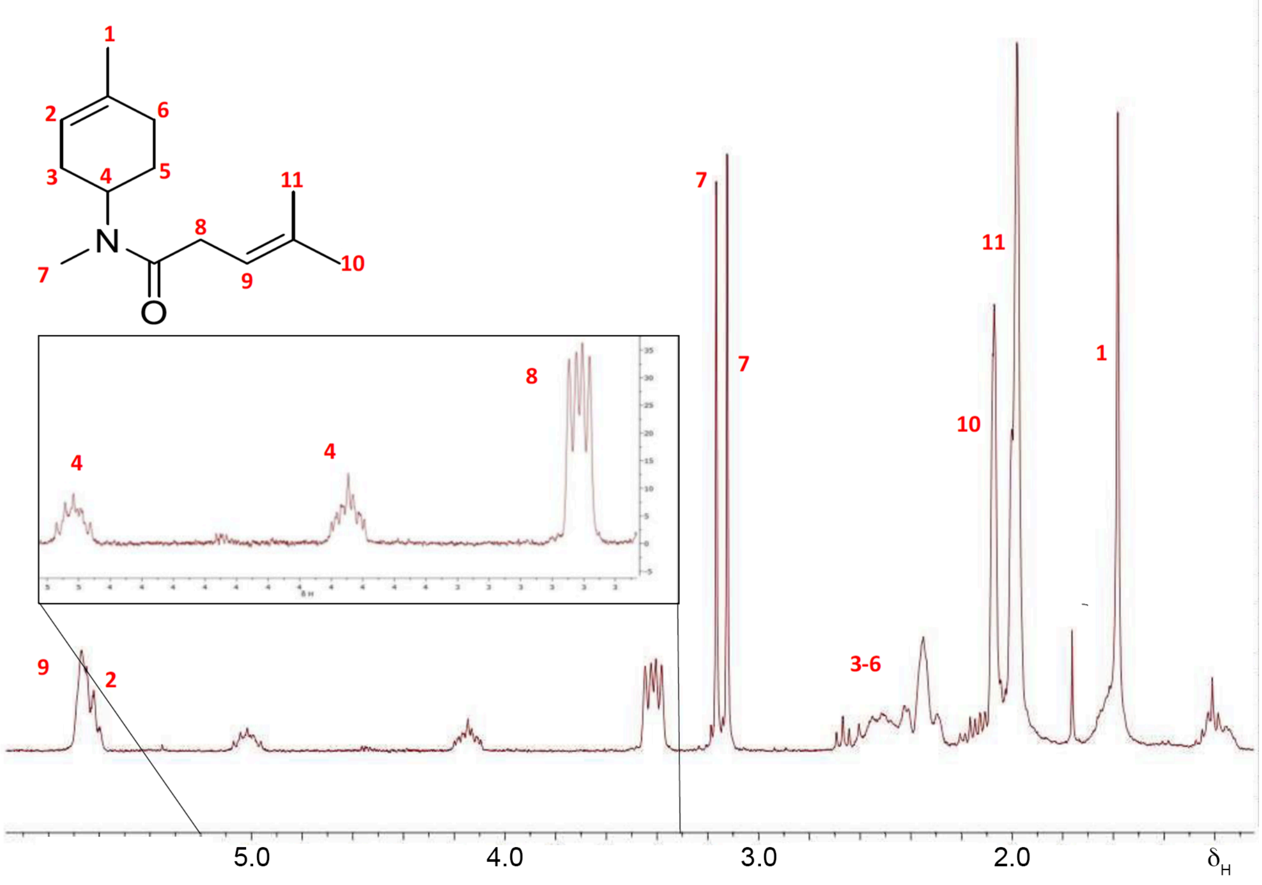

Figure **S30**. ^{1}H-NMR spectrum (300 MHz, $CDCl_3$) of **21**.

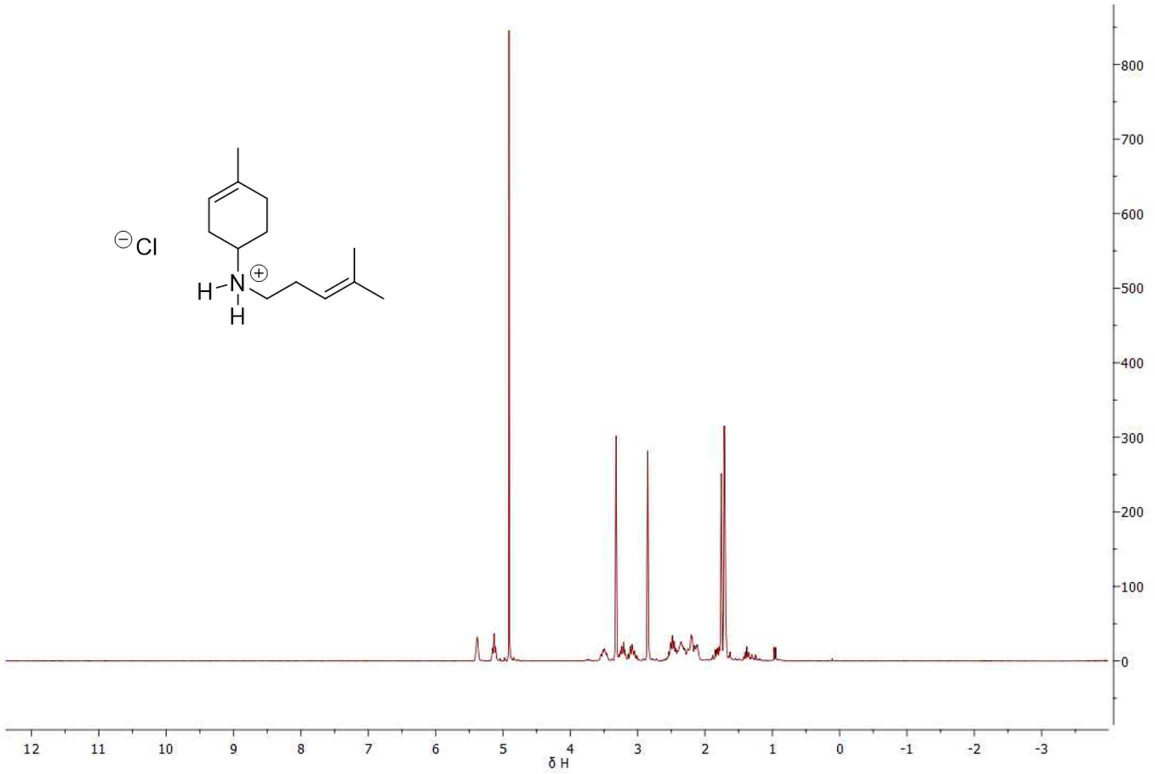

Figure S31. ^{1}H-NMR spectrum (300 MHz, $CDCl_3$) of **11**.

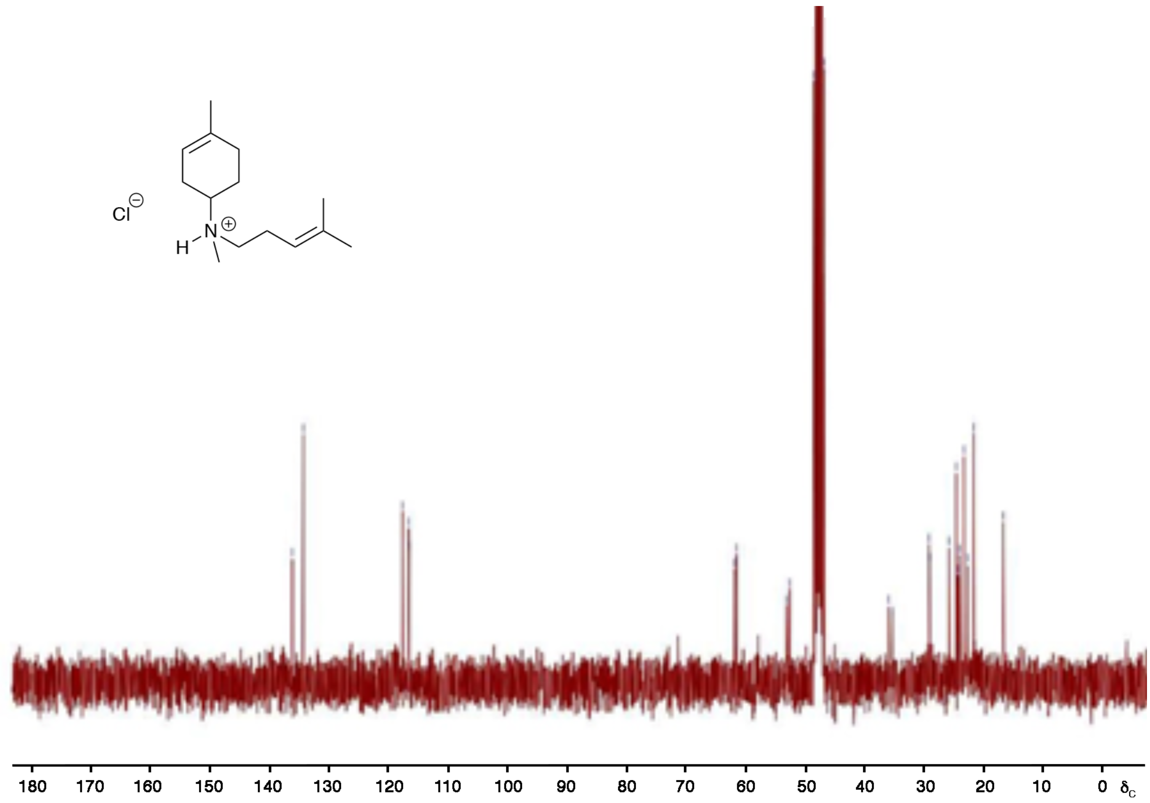

Figure S32. ^{13}C-NMR spectrum (300 MHz, $CDCl_3$) of **11**.

9.References

1 M. Loizzi. V. González, D. J. Miller, R. K. Allemann, *ChemBioChem* 2018, **19**, 100.

2 M. Demiray, X. Tang, T. Wirth, J. A. Faraldos, R. K. Allemann, *Angew. Chemie Int. Ed.* 2017, **56**, 4347.

3 S. Picaud, P. Mercke, X. He, O. Sterner, M. Brodelius, D. E. Cane and P. E. Brodelius, *Arch. Biochem. Biophys.*, 2006, **448**, 150.

4 V. J. Davisson, A. B. Woodside, T. R. Neal, K. E. Stremler, M. Muehlbacher and C. D. Poulter, *J. Org. Chem.*, 1986, **51**, 4768.

5 J. A. Faraldos, B. Kariuki and R. K. Allemann, *J. Org. Chem.* 2010, **75**, 1119.
